# Multi-omics analysis in primary T cells elucidates mechanisms behind disease-associated genetic loci

**DOI:** 10.1186/s13059-025-03492-y

**Published:** 2025-02-10

**Authors:** Chenfu Shi, Danyun Zhao, Jake Butler, Antonios Frantzeskos, Stefano Rossi, James Ding, Carlo Ferrazzano, Charlotte Wynn, Ryan Malcolm Hum, Ellie Richards, Muskan Gupta, Khadijah Patel, Chuan Fu Yap, Darren Plant, Richard Grencis, Paul Martin, Antony Adamson, Stephen Eyre, John Bowes, Anne Barton, Pauline Ho, Magnus Rattray, Gisela Orozco

**Affiliations:** 1 Centre for Genetics and Genomics Versus Arthritis, Division of Musculoskeletal and Dermatological Sciences, School of Biological Sciences, Faculty of Biology, Medicine and Health, The University of Manchester, Manchester, UK; 2 The Kellgren Centre for Rheumatology, Manchester Royal Infirmary, Manchester University NHS Foundation Trust, Manchester, UK; 3 Division of Immunology, Immunity to Infection and Respiratory Medicine, School of Biological Sciences, Faculty of Biology, Medicine and Health, The University of Manchester, Manchester, UK; 4 Genome Editing Unit, School of Biological Sciences, Faculty of Biology, Medicine and Health, The University of Manchester, Manchester, UK; 5 NIHR Manchester Biomedical Research Centre, Manchester University NHS Foundation Trust, Manchester Academic Health Science Centre, Manchester, UK; 6 Division of Informatics, Imaging and Data Sciences, Faculty of Biology, Medicine and Health, University of Manchester, Manchester, UK

**Keywords:** Chromatin conformation, Hi-C, Inflammatory diseases, Gene regulation, GWAS, QTLs

## Abstract

**Background:**

Genome-wide association studies (GWAS) have uncovered the genetic basis behind many diseases and conditions. However, most of these genetic loci affect regulatory regions, making the interpretation challenging. Chromatin conformation has a fundamental role in gene regulation and is frequently used to associate potential target genes to regulatory regions. However, previous studies mostly used small sample sizes and immortalized cell lines instead of primary cells.

**Results:**

Here we present the most extensive dataset of chromatin conformation with matching gene expression and chromatin accessibility from primary CD4^+^ and CD8^+^ T cells to date, isolated from psoriatic arthritis patients and healthy controls. We generated 108 Hi-C libraries (49 billion reads), 128 RNA-seq libraries and 126 ATAC-seq libraries. These data enhance our understanding of the mechanisms by which GWAS variants impact gene regulation, revealing how genetic variation alters chromatin accessibility and structure in primary cells at an unprecedented scale. We refine the mapping of GWAS loci to implicated regulatory elements, such as CTCF binding sites and other enhancer elements, aiding gene assignment. We uncover *BCL2L11* as the probable causal gene within the rheumatoid arthritis (RA) locus rs13396472, despite the GWAS variants’ intronic positioning relative to *ACOXL*, and we identify mechanisms involving *SESN3* dysregulation in the RA locus rs4409785.

**Conclusions:**

Given these genes’ significant role in T cell development and maturation, our work deepens our comprehension of autoimmune disease pathogenesis, suggesting potential treatment targets. In addition, our dataset provides a valuable resource for the investigation of immune-mediated diseases and gene regulatory mechanisms.

**Supplementary Information:**

The online version contains supplementary material available at 10.1186/s13059-025-03492-y.

## Background

Genome-wide association studies (GWAS) have uncovered a large proportion of the genetic basis underlying most common traits and diseases [1], such as psoriatic arthritis (PsA) [2] and rheumatoid arthritis (RA) [3]. It is widely established that these genetic associations are implicated mainly in the regulation of genes rather than altering coding sequences directly [4, 5]. Because regulatory elements perturbed by these variants can affect genes located far away from their genomic location with complex, cell-type specific, mechanisms [6–10], understanding the functional role of these variants involves significant effort and currently remains the main challenge in the field [11, 12].

Many studies have used functional genomics techniques to study risk loci, both to identify the causal variants among those in strong linkage disequilibrium (LD) and to assign candidate target genes [13]. These include using epigenomic markers to identify the variants that are likely to affect regulatory elements in disease-relevant cell types [7, 14], eQTL methods [15, 16], quantifying co-activation of enhancers and promoters [17, 18], and using chromatin conformation capture methods to assign enhancers to genes [19–30]. Most of these studies have, however, used cell lines or healthy subjects due to the difficulty of generating Hi-C libraries from primary cells and tissues, but there is extensive evidence that gene regulation and chromatin conformation are highly cell state specific [31–33]. These studies also typically tested small numbers of samples, and the only report examining the effect of natural DNA variation on 3D chromatin structure used cell lines and a technique with low resolution [34]. Finally, many of these studies used capture techniques, such as ChIA-PET [35], HiChIP [25], or capture Hi-C [19], which limit the view of the global chromatin structure.

Here we address these issues by generating the largest collection of high-quality, high-resolution, Hi-C maps in primary T cells using the commercial Arima Hi-C protocol, which allowed greater reproducibility whilst reducing the input material required, enabling the use of primary cells isolated from patients. We chose PsA as an exemplar disease as this autoimmune condition has a strong, complex genetic component and high heritability [36, 37]. Additionally, the mechanisms underlying this disease are not well understood and have not been the focus of previous functional genomics studies.

We interpret the results from our study using the latest understanding of how chromatin conformation is linked to gene regulation, validating recently published gene regulatory mechanisms [38–43]. We study how chromatin conformation varies across a population of individuals in different cell types and conditions and with genotype, at a significantly higher resolution to that previously achieved [34]. Finally, we show how this rich dataset allows us to decipher the mechanisms by which disease-associated variants increase the risk of developing disease by studying a selection of GWAS loci from a variety of immune conditions.

## Results

### A compendium of functional genomics data in primary T cells

**Fig. 1:**
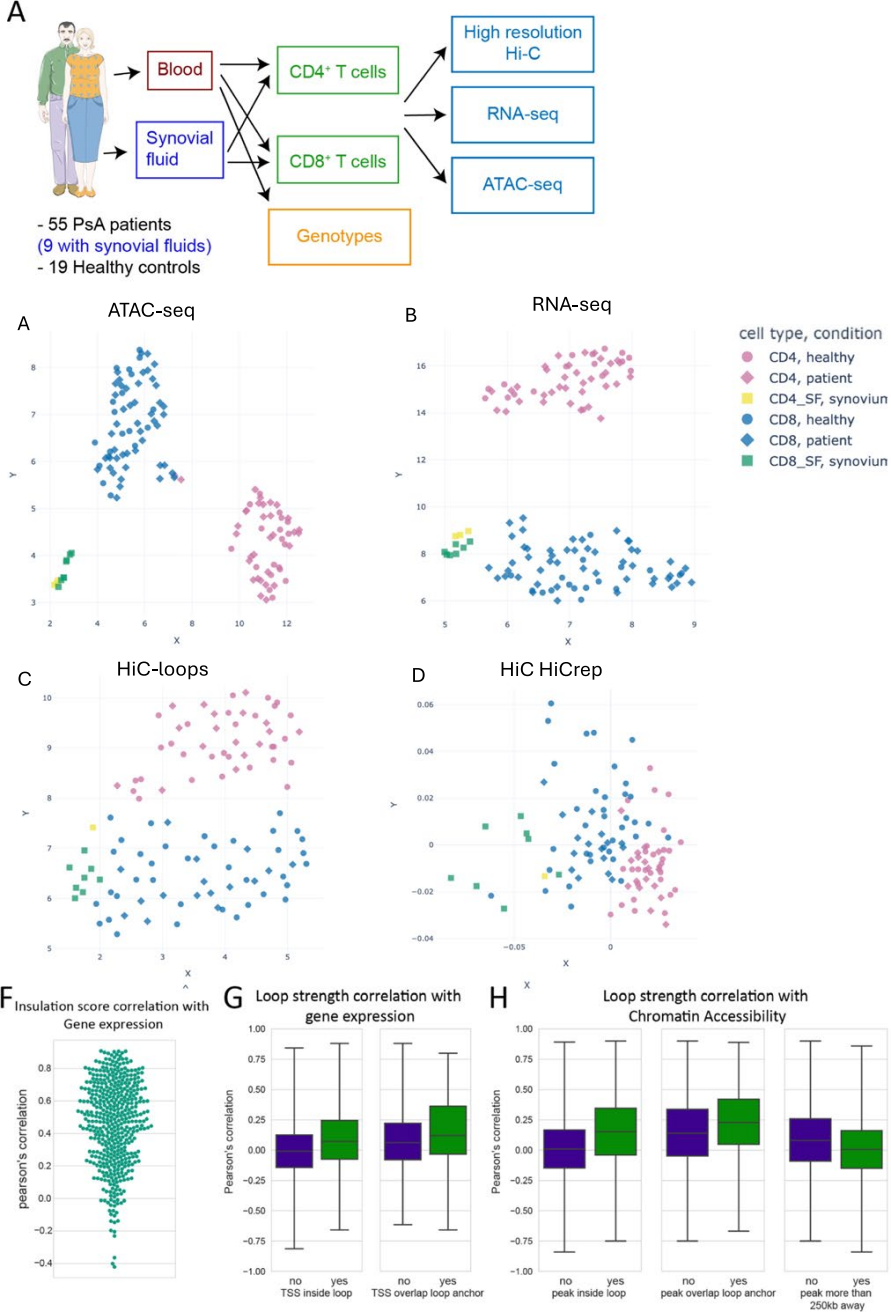
Study design, metrics, and correlation between modalities. **A** Summary of the study design. **B** UMAP of RNA-seq data, counts from DESeq2. **C** UMAP of the ATAC-seq data, counts from DiffBind. **D** UMAP of the Hi-C loops data. **E** Multidimensional scaling plot (MDS) of HiCRep analysis of the Hi-C data. **F** Pearson’s correlation between gene expression and insulation score of the bin overlapping promoter of the gene for genes that were differentially expressed between CD4 and CD8 cells. Each dot is a gene. **G** Pearson’s correlation between gene expression and loop strength. Left: comparison for loops that surround the gene vs loops that are nearby the gene. Right: comparison between loops that surround the gene with loops for which loop anchor overlaps the promoter of the gene. **H** Pearson’s correlation between the height of ATAC-seq peaks and loop strength. Left: Comparison between loops that surround the peak vs loops that are nearby the peak. Middle: Comparison between loops that surround the peak with loops for which loop anchor overlaps the peak. Right: Comparison between loops that are nearby the peak and loops that are more than 250 kb away

In this study, we present the largest dataset to date of matching chromatin conformation, chromatin accessibility, and gene expression from freshly isolated primary CD4^+^ and CD8^+^ T cells from 55 PsA patients and 19 Healthy controls from peripheral blood and, for a subset of patients, synovial fluid (Fig. 1A). This consists of 108 Hi-C libraries (49 billion reads), 128 RNA-seq libraries, and 126 ATAC-seq libraries. Uniform Manifold Approximation and Projection (UMAP) plots show that the samples are separated by cell type, with the synovial fluid samples forming a separate cluster from the peripheral blood samples (Fig. 1B–E). We do not detect a clear separation between samples derived from PsA patients and healthy controls (Fig. 1B–E), but we see a slight separation by sex of the individuals (Additional file 1: Fig. S1). Additionally, Euclidean distances between Hi-C replicates are significantly smaller than distances across patients, indicating that our library preparation method is able to detect inter-individual differences (Additional file 1: Fig. S2). MultiQC reports of common quality metrics and additional metadata for these datasets is available online http://bartzabel.ls.manchester.ac.uk/orozcolab/SNP2Mechanism/ and in Additional file 2: Table S1.

We identify chromatin loops using Mustache, a recently developed loop caller specifically designed for high-resolution contact maps [44]. We identified 69,303, 72,884, 30,415, and 12,000 loops from merged maps from CD4^+^, CD8^+^, CD8^+^ synovial fluid, and CD4^+^ synovial fluid T cells, respectively, which are then used for further analysis. As expected, 75% of these loops overlap a CTCF peak in at least one anchor, with stronger loops more likely to overlap a CTCF peak (Additional file 1: Fig. S3A–C), although only 42% overlap a CTCF peak at both anchors. Additionally, we find that 89% of loops overlap a chromatin accessibility peak at one end, 75% of loops overlap one at both ends and 47% of loops overlap a transcription start site (TSS).

Although it is known that immortalized cell lines differ significantly from primary cells, it has never been shown whether the chromatin conformation is altered. Here, we generated matching Hi-C libraries from two cell lines, Jurkat CD4^+^ T cells and MyLa CD8^+^ T cells to compare with our primary T cells datasets. Previous studies have shown that topologically associating domains (TADs) are generally stable between different cell types and differentiation states [45]. In line with these findings, we find that around 95% of TAD boundaries from MyLa and Jurkat cells are present in their corresponding primary cells and 99% of TAD boundaries from primary cells are present in the cell lines. For reference, two primary T cell samples share around 97% of TAD boundaries. However, we can identify large differences when looking at the compactness of these TADs. We estimate, using an outlier detection technique (see the “Methods” section), that 45% of the genome has significant differences in insulation score (Additional file 1: Fig. S3D). We also see large differences when we analyze high-resolution loops. We find that up to 20% of all loops are differentially interacting in cell lines compared to primary cells (Additional file 1: Fig. S3E). These regions with altered chromatin conformation include genes that are important in T-cell function such as the *ANKRD44*, *KLRF1*, *ITK*, and *GZMB* genes (Additional file 1: Fig. S4–5) and regions that overlap disease-associated GWAS loci such as the PsA loci rs11743851 (*SLC22A5*) or rs13203885 (*FYN*) (Additional file 1: Fig. S6).

### Chromatin conformation is highly associated with gene regulation

Next, we explored how chromatin conformation varies across the cell populations studied. Out of 105,956 loops, 29,454 were differentially interacting between CD8^+^ and CD4^+^ T cells (FDR < 0.1), and out of 113,350 insulation score bins at 25 kb, 41,614 bins have differential compactness. Of the 86,720 combined loops, 15,597 were differentially interacting between CD8^+^ T cells isolated from blood and synovial fluid (FDR < 0.1), together with 30,408 bins with differential compactness.

Although we could identify 166 (CD4^+^) and 38 (CD8^+^) genes differentially expressed between patients with active and non-active disease and 110 (CD4^+^) and 437 (CD8^+^) genes between cells from patients with active disease compared with cells from healthy subjects (Table 1), we could not identify any significant differential looping or differential insulation between these subgroups. This is likely because the differences between these subgroups are significantly smaller compared to the differences between cell types (Table 1). Additionally, patients and healthy controls may represent heterogenous groups as due to logistical and study design constraints these patients have been exposed to a variety of treatments and are at various levels of disease activity. We have carried out unsupervised clustering of the RNA-seq data from these patients in an attempt to identify more heterogenous groups. When clustering on samples using RNA-seq data within CD4^+^ and CD8^+^ T-cells, we identify 4 clusters for each cell type. However, our data does not show clear clustering on disease status (Additional file 1: Fig. S7). To integrate chromatin accessibility, we selected clusters of interest where differences between them were driven by immune-related pathways identified using KEGG (Additional file 1: Fig. S8) and conducted a differential accessibility analysis (clusters 1 vs 2 and 1 vs 3 for CD4^+^ T-cells, and clusters 1 vs 2 for CD8^+^ T-cells). We show that there are distinct chromatin accessibility patterns between clusters that correlate with gene expression clusters (Additional file 1: Fig. S9). Within each cluster, we found that the number of significant interactions between genes and differentially accessible peaks was significantly higher for differentially expressed genes compared to non-differentially expressed genes, suggesting that differentially accessible peaks regulate cluster-specific differential gene expression (Additional file 1: Fig. S10).

**Table 1:** Number of differentially expressed genes and differentially accessible ATAC-seq peaks

**Comparison**	**Differentially expressed genes**	**Differentially accessibl**e** ATAC-seq peaks**
Active vs non-active disease CD8^+^ T cells	38	3
Active vs non-active disease CD4^+^ T cells	166	1463
Active disease vs controls CD8^+^ T cells	437	127
Active disease vs controls CD4^+^ T cells	110	116
CD8^+^ vs CD4 + T cells	10109	43648
CD8^+^ SF vs CD8 + Active disease Blood	12709	30079
CD4^+^ SF vs CD4 + Active disease Blood	11536	37411

We next explored the interplay between gene expression and chromatin conformation. Our data shows a strong positive correlation between the insulation score of the chromatin domain and gene expression (Fig. 1F). Expanding on this finding, we examined the correlation between gene expression and the strength of the chromatin loops. As expected, we observed a strong positive correlation when a loop overlapped the gene promoter on one of the anchors. Interestingly, loops that encompassed a gene promoter, but not at the loop anchors, also showed correlation, albeit to a lesser extent (Fig. 1G). This indicates that indirect spatial relationships between promoters and loop anchors are associated with gene expression. Next, we included chromatin accessibility in the analysis. In this context, ATAC-seq peaks within a loop but that did not directly overlap the loop anchors exhibited a significant positive correlation with the loops, surprisingly even stronger than that observed for TSS (Fig. 1H). This supports the possible regulatory role of accessible chromatin regions, irrespective of their spatial relation to loop anchors, further extending our understanding of the influence of chromatin conformation on gene expression. These findings collectively suggest a nuanced role for chromatin architecture in transcriptional regulation.

**Fig. 2:**
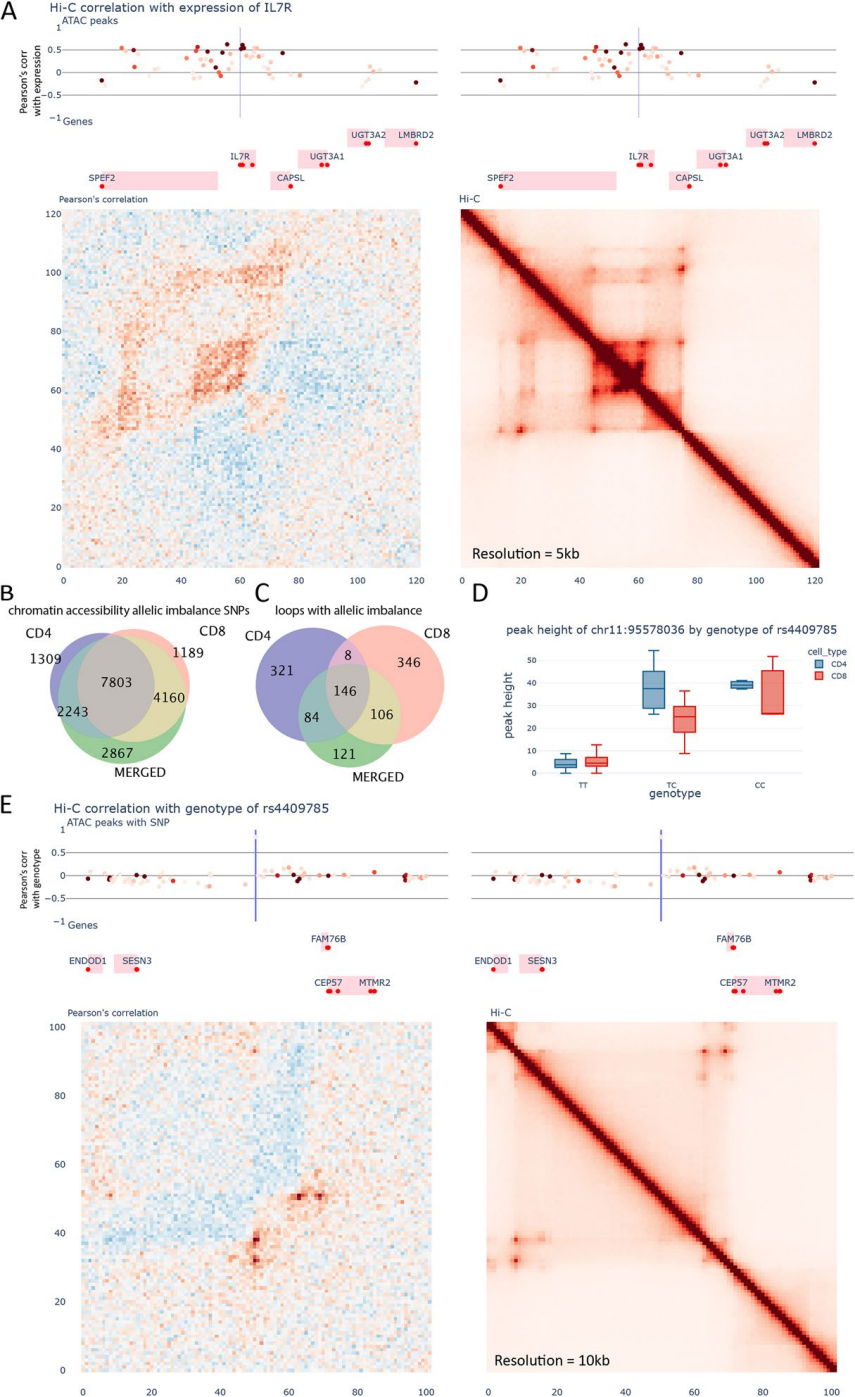
Chromatin conformation is linked to gene expression and genotype. **A** Visualization of the region surrounding *IL7R*. Top: ATAC-seq peaks. The intensity of the color indicates the average peak height of the peak, whilst position across the *Y*-axis indicates Pearson’s correlation with the expression of *IL7R*. Middle: Genes from Gencode v29. The red dots indicate the transcription start sites. Bottom left: Correlation between the Hi-C contacts and the expression of *IL7R*. Bottom right: Merged Hi-C map. **B** Venn diagram showcasing the overlap between chromatin accessibility allelic imbalance results from CD4^+^ T cells, CD8^+^ T cells, and merged dataset. **C** Venn diagram showcasing the overlap between loop allelic imbalance results from CD4^+^ T cells and CD8^+^ T cells. **D** Accessibility of the CTCF site located at chr11:95,578,036 vs the genotype of rs4409785. **E** Visualization of the region surrounding rs4409785. Top: ATAC-seq peaks. The intensity of the color indicates the average peak height of the peak, whilst position across the *Y*-axis indicates Pearson’s correlation with the genotype of rs4409785. Middle: Genes from Gencode v29. The red dots indicate the transcription start sites. Bottom left: Correlation between the Hi-C contacts and the genotype of rs4409785. Bottom right: Merged Hi-C map

Delving deeper into specific regions, our data enables us to visualize intricate local correlations in chromatin conformation. For instance, within the *IL7R* gene region (Fig. 2A), which is located in a large TAD also containing the *SPEF2* and *CAPSL* genes, we observed complex changes associated with fluctuating interactions around the subTAD encompassing *IL7R*. Here, compactness is negatively correlated with interactions outside of this subTAD, but positively correlated with interactions upstream to an intron of *SPEF2*. We find that the chromatin accessibility peaks located in this region are positively correlated with the expression of *IL7R*, suggesting a complex orchestration of chromatin interactions that regulate gene expression. We find that these complex changes in local chromatin conformation are very common across many important genes and our data can help understand the mechanisms involved in the regulation of these genes.

### Natural genetic variation is strongly associated with alterations in gene expression, chromatin accessibility, and chromatin conformation

Building upon these findings, we proceeded to assess the impact of common genetic variants on gene expression, chromatin accessibility, and chromatin conformation. We implemented insulation score QTL (insQTL) and loop strength QTL (loopQTL) in the context of chromatin conformation, to investigate the influence on the strength and insulation of topologically associating domains (TADs) and the strength of chromatin loops respectively. In conjunction, chromatin accessibility QTL (caQTL) was used to discern how genetic variation affects the functionality of enhancers and other regulatory elements and eQTL for gene expression. The number of significant loops, insulation score bins, chromatin accessibility peaks, and genes with a significant QTL are presented in Table 2. We find that a lot fewer QTLs were identified in the loopQTLs suggesting that loopQTL discovery may be more challenging than eQTLs and caQTLs due to subtler changes in chromatin conformation and sparser data.

**Table 2:** Number of significant QTLs (FDR < 0.10) for each of the methodologies tested (*n* = the number of samples that were used in the calculation after removal of outliers)

QTL methodology	Number of significant QTLs (phenotypes)
CD8^+^ T cells eQTL (*n* = 64)	1609
CD4^+^ T cells eQTL (*n* = 47)	1015
CD8^+^ T cells caQTL (*n* = 65)	7861
CD4^+^ T cells caQTL (*n* = 49)	6082
CD8^+^ T cells insQTL (*n* = 53)	7180
CD4^+^ T cells insQTL (*n* = 46)	8284
CD8^+^ T cells loopQTL (*n* = 53)	579
CD4^+^ T cells loopQTL (*n* = 44)	762

Next, we explored how these different QTL results intersect with one another. Initially, we compared the results from the same modality across the two cell types (CD8^+^ and CD4^+^ T cells). We find that these QTLs are highly concordant between the two cell types studied with about 75–85% of QTLs significant (FDR < 0.10) in one cell type having at least an uncorrected p-value of less than 0.01 in the other cell type (Additional File 3:Table S2) and with essentially 100% of these having the same directionality (Additional file 1: Fig. S11).

Continuing our investigation, we examined how QTLs from different modalities overlapped each other. We could recover approximately 38% of eQTLs from the caQTLs that overlapped the promoters of the eQTL genes, with a concordance rate of 88%. Similar levels of overlap and concordance were found between eQTLs and insQTLs and caQTLs and insQTLs. These findings are in line with the results of our previous section, corroborating the notion that stronger gene expression is associated with increased chromatin domain insulation.

When we considered the overlap between eQTLs and caQTLs with loopQTLs, the concordance dropped to 70% and 78%, respectively, if we included all loops for which the promoter or the ATAC-peak was located between the two loop anchors. However, the concordance increased, reaching 85% and 91% respectively, when we included only the loops that overlapped the promoter or the ATAC-peak at loop anchors. This suggests that loops that directly overlap the promoter of the genes or the ATAC-peak are more likely to be positively correlated with the expression of the genes and the activity of the peak. Conversely, loops that do not overlap the promoter or the ATAC-peak, whilst still significant QTLs, do not necessarily have a positive correlation, indicating possibly different mechanisms at play.

An alternative method to study the effect of a genetic variant with a molecular phenotype is allelic imbalance. This approach calculates the proportion of signal derived from each allele in heterozygous individuals and can also be helpful in pinpointing the causal variants among those in LD. Because a large proportion of GWAS SNPs are predicted to affect enhancers, we applied this technique to chromatin accessibility regions to identify enhancers altered by these variants. We identified 19,571 variants with allelic imbalance in chromatin accessibility (*S*-value < 0.1) out of 243,664 variants overlapping open chromatin regions in the merged dataset (Fig. 2B). We found that allelic imbalance produces slightly different results and is slightly more sensitive than QTL analysis (see methods for more information); For the variants that overlap between the two analyses, we find 98% concordance in their directionality. Additionally, we have applied this technique to our chromatin conformation data, identifying 1132 loops with allelic imbalance across the two cell types (Fig. 2C). In this case, we find that only 5% of the loopQTLs have an associated variant within their loop anchors and most of the loops don’t have enough sequencing depth to call allelic imbalance significantly (albeit with 100% concordance in directionality for the significant ones).

Next, we use these results to interrogate a different set of mechanisms to increase our understanding of GWAS loci.

### Unveiling the consequence of GWAS variants that affect the binding of CTCF

One of the most important proteins in the regulation of chromatin architecture is CTCF. For this reason, we searched for variants within CTCF binding sites in the allelic imbalance loops and loopQTLs. We found that 18% of loops with allelic imbalance have one variant located within a CTCF peak and that 45% of loopQTLs and 22% of insQTL have at least one variant overlapping a CTCF peak. These results indicate that even though a majority of these structures are defined by CTCF-related mechanisms, the way genetic variants affect these interactions may not always be driven by alteration of the CTCF binding. Interestingly, we find that about 20% of GWAS loci have at least one variant overlapping a CTCF peak. We tested for enrichment of GWAS variants in CTCF peaks using fGWAS, for a number of autoimmune diseases where T cells play an important role and for which summary statistics were available (PsA, RA, JIA). This analysis indicated no significant enrichment (Additional File 4:Table S3). However, although this may not be the predominant mechanism by which most GWAS variants cause disease, we find examples were specific GWAS loci affect CTCF peaks. For example, in the RA locus tagged by rs4409785, the lead SNP is a strong caQTL for the peak it maps to (FDR 7.91e − 4) (Fig. 2D). Previous studies have revealed that the risk allele of this SNP creates a new CTCF binding site [46]. Our data supports this previous finding and suggests how the creation of this new CTCF binding site could alter the local chromatin structure (Fig. 2E). This SNP is located at the center of a large chromatin domain, which contains the gene *SESN3* and a large gene desert. The risk allele of rs4409785 is associated with the creation of two new loops, not visible in the merged high-resolution Hi-C, which connect this element to the downstream boundary of the chromatin domain. This results in an increase of the contacts within this new subTAD, and a reduction of interactions between this region and the upstream region which includes the *SESN3* gene, thereby reducing the ability of regulatory elements positioned downstream of rs4409785 to activate the gene *SESN3*. Public eQTL data from eQTLgen shows that the risk allele of rs4409785 is associated with a reduction of expression of *SESN3* [15]. This risk locus had been previously assigned to the *CEP57* gene [3]. However, *SESN3* could have an important role in RA. A recent study suggests that SESN3^+^ memory T cells, a subset not extensively studied before, may play a significant role in the development of arthritis or autoimmune diseases. It was found that these cells, which differentially express another key RA gene, *TNFAIP3*, could differentiate into cytotoxic CD4^+^ T cells, potentially contributing to the pathogenesis of autoimmune diseases [47].

**Fig. 3:**
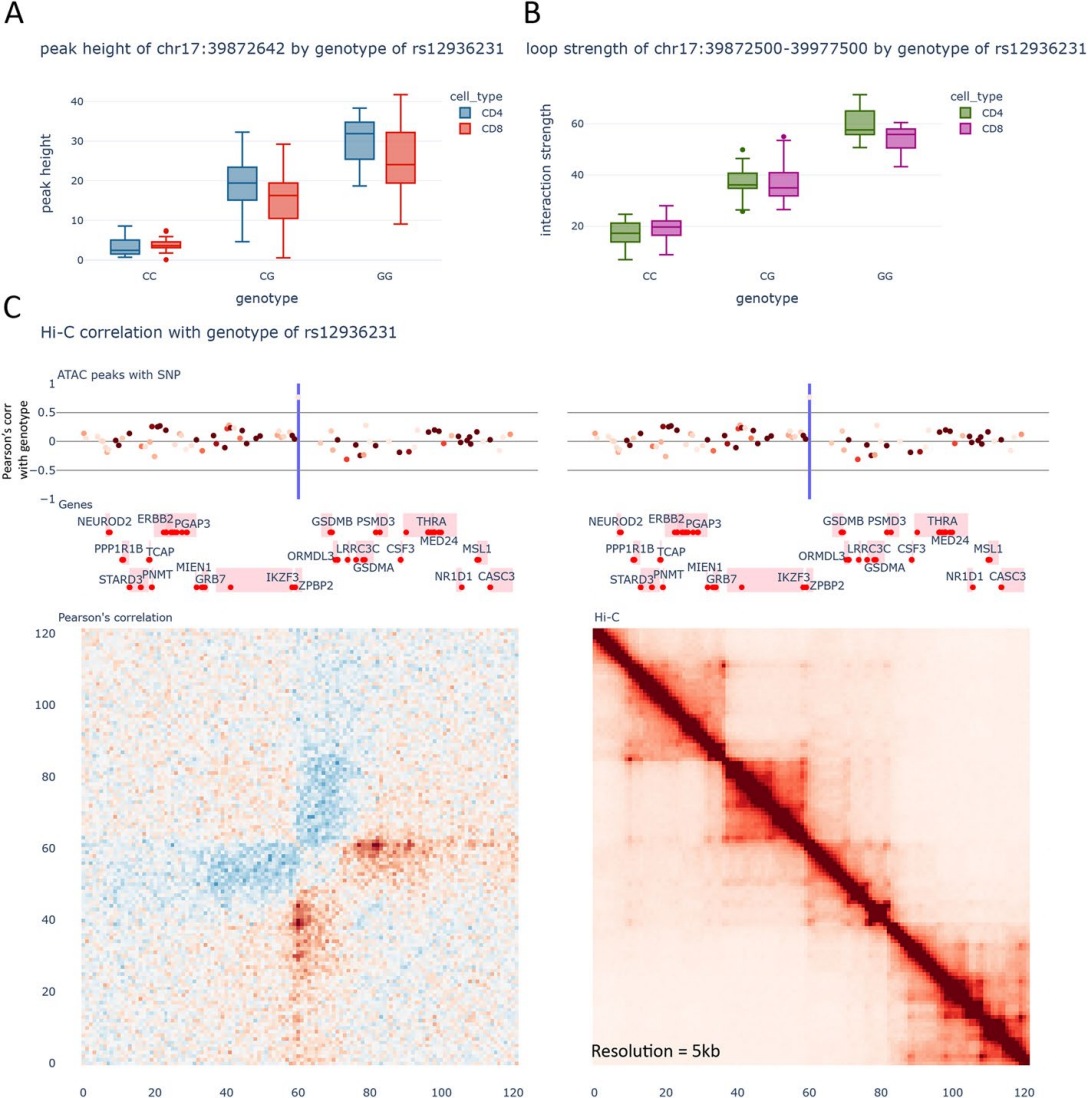
Gene regulatory mechanisms underlying the rs12936231 locus. **A** Accessibility of the CTCF site located at chr17:39,872,642 vs the genotype of rs12936231. **B** Representative loopQTL with the genotype of rs12936231 in the region. Loop displayed is chr17:39,872,500–39977500. **C** Visualization of the region surrounding rs12936231. Top: ATAC-seq peaks. The intensity of the color indicates the average peak height of the peak, whilst position across the *Y*-axis indicates Pearson’s correlation with the genotype of rs12936231. Middle: Genes from Gencode v29. The red dots indicate the transcription start sites. Bottom left: Correlation between the Hi-C contacts and the genotype of rs12936231. Bottom right: Merged Hi-C map

Another interesting example is the *IKZF3*/*GSDMB*/*ORMDL3* GWAS locus, which is linked to many autoimmune diseases, including RA, asthma, systemic sclerosis (SSc), and others [3, 48, 49]. Previous studies have localized the causal SNP as rs12936231 [46, 50, 51], which creates a novel CTCF binding motif. We found in our dataset that this SNP is a strong caQTL for the ATAC-peak (FDR 4.03e − 4 for CD4 and 6.19e − 8 for CD8) (Fig. 3A) and displays strong loop allelic imbalance (*S*-value 6.27e − 8 for CD4 and 1.28e − 12 for CD8) and multiple loopQTLs (one example plotted in Fig. 3B). Although it is known that this novel CTCF binding site causes a change in chromatin looping, using our dataset, we can visualize the effect on this locus at an unprecedented detail. We find significant changes in the insulation of two contact domains (subTADs), associated with a reduction of interactions between them, whilst significantly increasing the interactions downstream of the new CTCF site (Fig. 3C). Our allelic imbalance data also shows that other variants upstream of rs12936231, and in LD with it, have increased chromatin accessibility which indicates that this reduction of interactions results in an increase in activity of these enhancers. Together with eQTL data, which indicate upregulation of genes upstream of the CTCF motif (*IKZF3*) and downregulation of genes downstream (*GSDMB*, *ORMDL3*), we can conclude that this variant increases disease risk by disrupting the ability of upstream regulatory elements to contact these downstream genes.

Another region with a similar mechanism is a locus associated with white blood cell count and neutrophil counts indexed by the SNP rs10424217. We find that the SNP in LD rs2353678, directly overlaps a predicted CTCF binding motif, with our data showing a strong increase in chromatin accessibility both through allelic imbalance (*S*-value < 1e − 308) and caQTL (FDR 1.53e − 9) (Additional file 1: Fig. S12A). The new potential CTCF site increases the separation between two chromatin domains (Additional file 1: Fig. S12B) and is associated with an increased expression of most genes in the region, including *RPS16* (upstream), *SUPT5H*, *EID2*, *EID2B*, *TIMM50* (downstream), but a decrease of expression of *PLEKHG2* (upstream).

In summary, we show how some variants can affect the regulation of multiple genes, by affecting key regulatory elements that define chromatin architecture at an unprecedented detail and resolution. However, the majority of the GWAS loci do not act through this mechanism. We explore how our data can help us investigate GWAS loci with other mechanisms in the following section.

### Uncovering the mechanisms by which GWAS loci affect gene regulation by altering enhancer function

Previous studies have indicated that the majority of GWAS loci affect enhancer elements [4]. We utilized our dataset to investigate the mechanisms involved behind this class of loci by first using chromatin accessibility allelic imbalance as a tool to functionally fine-map GWAS loci (Additional File 5: Table S4). Our analysis led to the identification of one or more allelic imbalance SNPs in about a third of the loci. For instance, in the *KLF13* psoriasis locus indexed by rs28624578, out of the 4 SNPs in LD, 3 overlap chromatin accessibility regions. However, our data shows that only rs28510484 exhibits strong allelic imbalance (*S*-value 1e − 6) with a 2.2-fold increase in chromatin accessibility. These SNPs are located in an intron of *KLF13* and eQTLgen data [15] show an increase in *KLF13* expression associated with the risk allele of rs28510484.

**Fig. 4:**
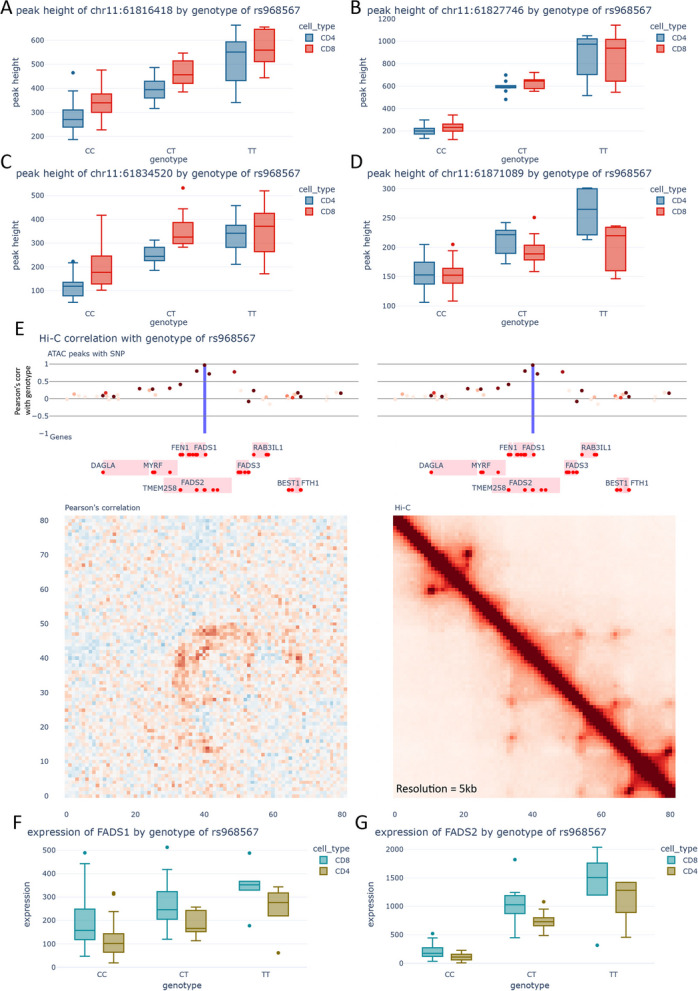
Gene regulatory mechanisms underlying the rs968567 locus. **A** Chromatin accessibility of peak chr11:61,816,418 vs the genotype of rs968567. **B** Chromatin accessibility of peak chr11:61,827,746 vs the genotype of rs968567. **C** Chromatin accessibility of peak chr11:61,834,520 vs the genotype of rs968567. **D** Chromatin accessibility of peak chr11:61,871,089 vs the genotype of rs968567. **E** Visualization of the region surrounding rs968567. Top: ATAC-seq peaks. The intensity of the color indicates the average peak height of the peak, whilst position across the *Y*-axis indicates Pearson’s correlation with the genotype of rs968567. Middle: Genes from Gencode v29. The red dots indicate the transcription start sites. Bottom left: Correlation between the Hi-C contacts and the genotype of rs968567. Bottom right: Merged Hi-C map. **F** Expression of *FADS1* with the genotype of rs968567. **G** Expression of *FADS2* with the genotype of rs968567

Remarkably, certain regions also display alterations in chromatin conformation. The RA locus indexed by SNP rs7943728 serves as an example, wherein the SNP in LD rs968567 is the only SNP that overlaps chromatin accessibility regions and displays pronounced allelic imbalance accompanied by an 8.2-fold increase in chromatin accessibility (*S*-value < 1e − 308) for the protective allele. This SNP is a strong caQTL for 4 separate ATAC-peaks, indicating that the alternate form of rs968567 causes an increased activity of other regulatory elements in the locus as well (Fig. 4A–D). This strong increase in the activity of these elements results in the increase in interactions in the local chromatin architecture, particularly those bringing the genes *FADS2*, *FADS1*, *FADS3*, and *FTH1* into closer contact with the enhancer elements affected by rs968567 (Fig. 4E). Moreover, the variant is correlated with an increase in the expression of *FADS2* and *FADS1* (Fig. 4F–G). These genes are crucial for the pathogenesis of RA as they play a key role in the biosynthesis of long-chain polyunsaturated fatty acids, like omega-3 and omega-6 [52], known to influence inflammation and immune response mechanisms pivotal in the disease's pathogenesis [53].

**Fig. 5:**
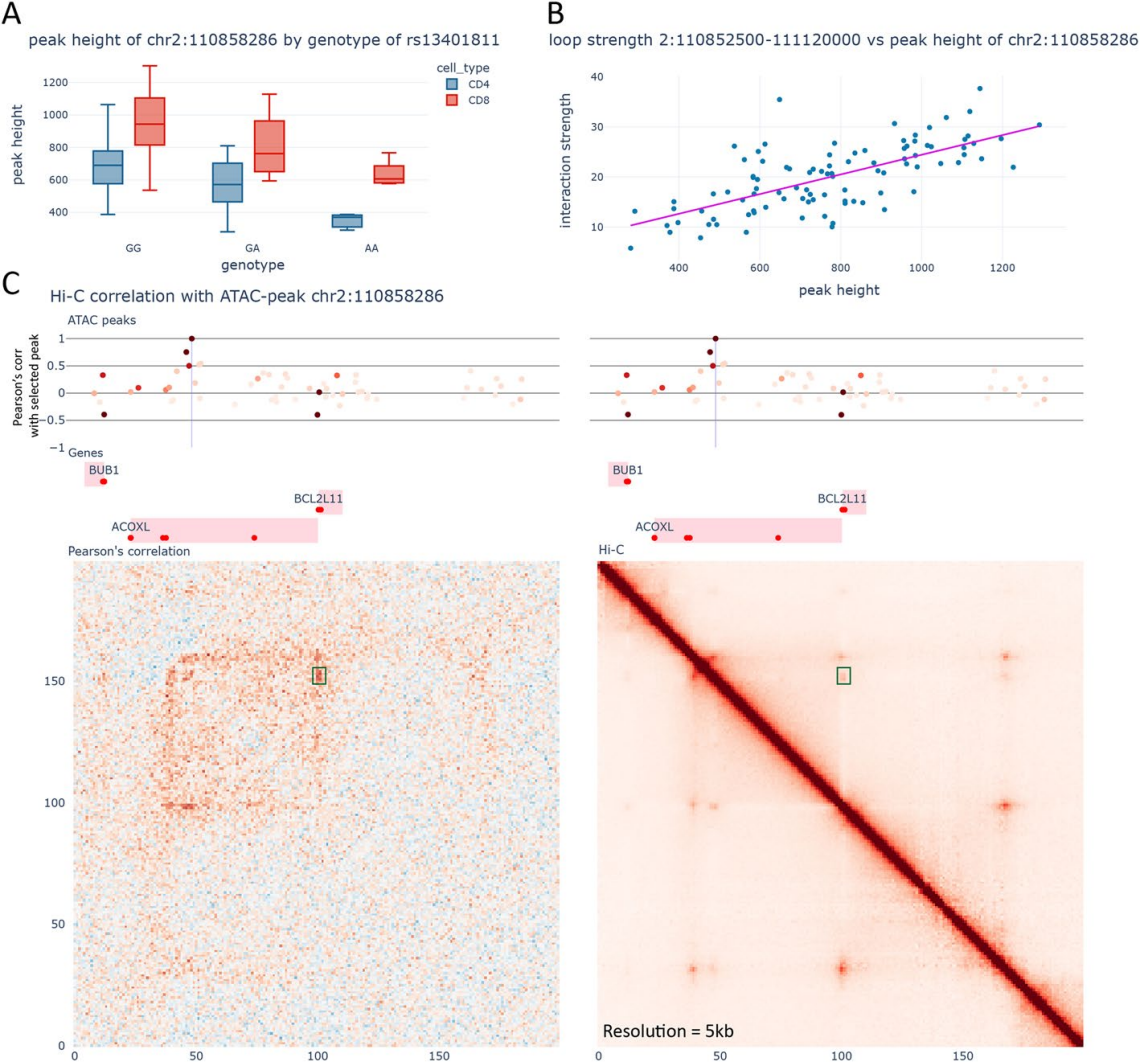
Gene regulatory mechanisms underlying the rs13401811 locus. **A** Chromatin accessibility of peak chr2:110,858,286 vs the genotype of rs13401811. **B** Loop strength of the loop connecting the enhancer with the promoter of *BCL2L11* vs the peak height of the enhancer. **C** Visualization of the region surrounding rs13401811. Top: ATAC-seq peaks. The intensity of the color indicates the average peak height of the peak, whilst position across the *Y*-axis indicates Pearson’s correlation with the peak height of the enhancer chr2:110,858,286. Middle: Genes from Gencode v29. The red dots indicate the transcription start sites. Bottom left: Correlation between the Hi-C contacts and the peak height of the enhancer chr2:110,858,286. Bottom right: Merged Hi-C map. The green box indicates the loop that connects the enhancer with the promoter of *BCL2L11*

Lastly, in the RA locus indexed by rs13396472, only one SNP, rs13401811, shows allelic imbalance (*S*-value 2.63e − 2), with a reduction of 40% in chromatin accessibility associated with the protective allele. This SNP is also a strong caQTL for the overlapping ATAC-seq peak (FDR 6.26e − 3, Fig. 5A). This locus has been previously linked to *ACOXL* because the SNP in LD rs1554005, is a missense variant for *ACOXL*. Additionally, rs13401811 is located in an intron of *ACOXL*. However, the function of *ACOXL* is not especially relevant for RA pathogenesis and it is not expressed in T cells (less than 5 reads in our data) or other immune cells according to the DICE database [16]. Our chromatin conformation data reveals a loop connecting the enhancer affected by rs13401811 to the promoter of *BCL2L11* a gene located more than 300 kb downstream of the GWAS SNPs. In fact, the activity of this enhancer appears to be highly correlated with the strength of the loop (p-value 1.25E − 15, R2 0.47, Fig. 5B–C). To validate this observed link, we employed CRISPR/Cas9 in primary human CD4 + T cells to delete approximately 200 bp encompassing the rs13401811 ATAC-seq peak. We examined the change in expression of *BCL2L11* following a 4-h stimulation with CD3/CD28 beads. We observed a marked decrease in the upregulation of *BCL2L11* in the CD4 + T cells following stimulation (*p*-value 0.008) in the enhancer knock-out T-cells compared to wild-type T-cells (Additional file 1: Fig. S13). This result supports the role of the rs13401811 enhancer region in regulating *BCL2L11* expression CD4 + T cells. *BCL2L11* has a critical role within the immune system, acting as a pro-apoptotic stimulator and modulating thymic negative selection. Knockout mice for *BCL2L11* display progressive autoimmune disease [54]. A knockout of the regulatory locus containing this SNP has been previously reported in mice [55]. These mice display altered expression of *BCL2L11* in specific T cells subsets, further strengthening our finding that rs13401811 increases the risk of developing RA by altering activation of *BCL2L11* expression.

## Discussion

In this study, we have produced the most extensive dataset to date combining chromatin conformation, chromatin accessibility, and gene expression data from primary T cells isolated from PsA patients and healthy individuals. Our data allowed us to clearly distinguish T-cell samples into the different populations. However, we found that chromatin conformation variations between patients and controls were subtle and hard to detect, in line with previous attempts in the field [56].

Our work corroborates the recent findings about gene regulatory mechanisms and the relationship between chromatin conformation and gene regulation [38–43]. In that regard, we observed a strong correlation between chromatin compactness, as measured by insulation score, and gene expression, alongside looping strength. Additionally, our dataset enabled us to discern subtle alterations in local chromatin conformation linked to genotype, gene expression, and chromatin accessibility peaks, thus revealing intricate dynamics that remain hidden in conventional merged data.

We also present the most extensive analysis to date on the impact of genetic variation on chromatin conformation at an unprecedented resolution. By using complementary results from chromatin accessibility and gene expression we illustrate how variants that increase gene expression and chromatin accessibility also increase chromatin compactness and have intricate relationships with chromatin loops. Our results, however, indicate that our sample size is not sufficiently large to detect all QTLs, highlighting the ongoing need for larger-scale studies in this domain.

Finally, we leveraged our data to functionally refine GWAS loci. The interpretation of risk variants from GWAS has often been a stumbling block in their application to drug development. Compared to other studies, ours provides the most comprehensive functional context around these variants and underlines the complex nature of the mechanisms by which GWAS loci can influence gene regulation. The majority of prior studies relied on simple chromatin loops or raw contact frequency to associate genes with enhancers [19–22, 24–30, 57]. However, this approach poses several challenges. Firstly, the majority of chromatin loops observed in Hi-C maps are structural loops mediated by CTCF, which do not link promoters with enhancers. Moreover, the majority of chromatin conformation methods are bulk methods, which means that enhancer-promoter interactions that appear only in a subpopulation of the cells, or that are more transient [58], might be significantly harder to detect. In that regard, our correlation method allows for the identification of dynamic structures and changes in interaction frequency that are not visible in the merged Hi-C map, such as those depicted in the *FADS1/2* locus. Other techniques, for example, those based on Micro-C [39, 41, 42] have lower background noise compared to Hi-C but are significantly more challenging technically, requiring a large number of cells, and, to our knowledge, no study to date has been published that uses primary cells. Furthermore, difficulties in the reproducibility of the libraries limit the kind of correlation and QTL analysis that have been presented here. Secondly, focusing on enhancer-promoter interactions overlooks the exploration of other mechanisms by which genetic variants impact gene regulation and chromatin conformation, such as the CTCF binding altering variants presented in our study. These regions represent just the beginning of understanding the intricate mechanisms at play, such as the ability of specific variants to affect multiple genes at once and for regulatory elements to affect genes across TAD boundaries. This highlights the need to move beyond strict, static definitions of TADs, loops, and correlations in assigning genes to GWAS loci. Additionally, we identified notable differences between primary cells and cell lines, which suggest that caution is needed when using cell lines to draw conclusions. In this regard, further studies investigating CD4 + and CD8 + cell subtypes are needed to fully characterize the regulatory landscape of T-cells.

The mechanisms and genes we identified here through our dataset, such as *BCL2L11* and *SESN3*, present promising targets for novel, more effective therapeutics, and aid in patient stratification. New treatments that have genetic support are twice as likely to be approved [59]. *BCL2L11* is a key player in the apoptosis pathway and fundamental for T cell maturation [54, 55]. Methotrexate, a widely used drug in rheumatology, has an inhibitory effect on dihydrofolate reductase (*DHFR*) which can influence various pathways, including those involving apoptosis where *BCL2L11* plays a significant role. In this context, our findings about *BCL2L11* regulation or expression could provide novel avenues for future research, including the exploration of how variations in *BCL2L11* regulation might affect therapeutic outcomes or the development of new treatments that target *BCL2L11* directly. Another breakthrough from our study is the identification of the potential mechanism by which the RA locus rs4409785 affects the expression of *SESN3*, a gene that plays a pivotal role in a specific subset of T cells implicated in the pathogenesis of autoimmune diseases [47]. This discovery suggests the potential for novel therapies that target these cells directly.

## Conclusions

In conclusion, our research elucidates the complex dynamics of chromatin conformation and gene regulation, offering potential implications for disease understanding and therapeutic development. We anticipate that our dataset will be a valuable resource for researchers in this field. To facilitate further research, we have made available all pre-processed data, including precomputed chromatin conformation maps, correlations with gene expression, chromatin accessibility, genotype, QTL datasets, and code to replicate our analysis and annotate further GWAS results at http://bartzabel.ls.manchester.ac.uk/orozcolab/SNP2Mechanism/

## Methods

### Sample collection and cell isolation

Between 40 and 100 ml of peripheral blood was collected following informed consent from 55 PsA patients and 19 healthy controls and processed on the same day. Patients with a clinical diagnosis of PsA made by a consultant rheumatologist were eligible to be recruited. Physician global assessment (a Likert scale from 1 to 5, where a score of 1 indicates non-active disease and a score of 2–5 indicates active disease, with a score of 5 being the most severe disease activity). For the CRISPR/Cas9 experiments, buffy coats from 6 healthy donors were acquired from the National Health Service blood service. PBMCs were isolated using Ficoll density gradient centrifugation. EasySep Human CD4^+^ and CD8^+^ T cell negative selection kits (StemCell technologies #17,952 and #17,953) were used to isolate CD4^+^ and CD8^+^ T cells respectively. Cells were then aliquoted and prepared for long-term storage separately for each technique used.

Synovial fluid extracted from patients with PsA was first treated with hyaluronidase enzyme (Sigma-Aldrich #H3884) and then processed in a similar way as the peripheral blood samples.

To confirm that there was no sample mislabelling the concordance of the genotype with the bam file was checked using the QTLtools v1.3.1 mbv function [60]. To identify mismatches between CD4^+^ T cells and CD8^+^ T cells we used the loop strength of the loops surrounding the *CD4* and *CD8A* genes for the Hi-C data (Additional file 1: Fig. S14A–B), the gene expression of the *CD4* and *CD8A* genes for the RNA-seq data (Additional file 1: Fig. S14C–D), the chromatin accessibility of the promoters of *CD4* and *CD8A* for ATAC-seq (Additional file 1: Fig. S14E–F). Sample mismatches were then corrected in the metadata files.

To assess whether our Hi-C libraries could be sensitive enough to detect the interindividual differences we wanted to show, we have analyzed some limited replicate data for some libraries that were repeated due to failing QC. We have calculated the Euclidean distance between samples, by first gathering the counts for loop strength, running a PCA (as implemented in sklearn), and then calculating the Euclidean distance (as implemented in scipy) on the top 10 components, and we can show that Hi-C libraries generated from the same sample have a smaller distance than different libraries, even considering the low quality of these libraries (Additional file 1: Fig. S2A). In addition, direct hierarchical clustering of the Euclidean distances was performed using Ward’s linkage method (as implemented in scipy.cluster.hierarchy), and the resulting dendrogram demonstrates that replicate Hi-C libraries cluster closely, confirming the sensitivity of our approach in distinguishing between individual samples (Additional file 1: Fig. S2B) For RNA-seq and ATAC-seq, unfortunately, we did not have enough replicate data of reasonable quality to run this analysis. Additionally, we have calculated the distances between samples of the same cell type and the rest, and we can see that for all three modalities, the distances between two samples of the same cell type are much smaller compared to samples of different cell types (Additional file 1: Fig. S2C–E).

### Hi-C library preparation and sequencing

Approximately 3–5 million cells were crosslinked in 2% formaldehyde for 10 min at room temperature and the reaction was then quenched using a solution of glycine + BSA. Samples were then washed in PBS and snap frozen on dry ice and then transferred to − 80 °C for long-term storage.

Two million cells were then used for library preparation following the two-restriction enzyme Arima Hi-C kit (Arima Genomics) and the KAPA HyperPrep kit (Roche #KK8504) manufacturer’s protocol using Illumina TruSeq dual indexes.

Library size was checked by TapeStation 4200 (Agilent) and QC was done by quantstudio (Life technologies) using the NEBNext® Library Quant Kit for Illumina (E7630). Sequencing was performed on the NovaSeq6000 platform using NovaSeq S4 flow cells at a target depth of 350 million reads per library and generating 150 bp paired-end reads.

### RNA-seq library preparation and sequencing

Approximately 500 thousand to 1 million cells were lyzed and stored in 700 μL Qiazol lysis reagent (QIAGEN, ref: 79,306). To isolate RNA, 140 μL of chloroform was added. After centrifugation at 12,000 × g for 15 min, approximately 350 μL of the upper layer containing the RNA was transferred and mixed with 350 μL of 70% ethanol. RNA isolation was continued from this point using the RNeasy MinElute kit (QIAGEN, ref: 74,204) protocol. RNA libraries were then generated by NovoGene UK using a custom protocol based on cDNA synthesis using random hexamer primers and polyA mRNA enrichment. Sequencing was performed on the NovaSeq6000 platform using NovaSeq S4 flow cells at a target depth of 40 million reads per library and generating 150 bp paired-end reads.

### ATAC-seq library preparation and sequencing

Approximately 500 thousand cells were frozen in Recovery™ Cell Culture Freezing Medium (Thermofisher #12,648,010) and stored at -80 °C. An aliquot of 50 thousand cells was then used for ATAC-seq library preparation following the Omni-ATAC protocol as released by the Kaersten lab [61, 62].

Library size was checked by TapeStation 4200 (Agilent) and QC was done by quantstudio (Life technologies) using the NEBNext® Library Quant Kit for Illumina (E7630). Sequencing was performed on the NextSeq2000 platform at a target depth of 50 million reads per library and generating 50 bp paired-end reads.

### Hi-C data processing, loop calling, and clustering

Hi-C reads [63] were quality filtered and trimmed and adapters were removed using fastp v0.20.1 [64]. Reads were then processed and mapped to the GRCh38 genome using HiC-pro v3.0.0 [65], using default settings. Hic juicebox files for visualization and analysis were generated using the hicpro2juicebox.sh script using juicer tools v1.22.01. Cool files were generated from the juicebox files using hic2cool (https://github.com/4dn-dcic/hic2cool). TAD calling was done using onTAD [66] with default settings and ICE-normalized 40 kb resolution maps. Visualizations were made using a modified version of the python package coolbox [67] or through custom scripts. The Hi-C processing pipeline is available on GitHub [68] and Zenodo [69] (BSD 3-Clause License).

Loop calling was performed on merged libraries by cell population (CD4^+^, CD8^+^, and CD8^+^ synovial fluid) using Mustache [44] at a resolution of 2.5 kb and SCALE normalization. This tool allows the detection of locally enriched loops (similar to HICCUPS [70]) rather than defining a model of contact decay based on genomic distance (such as FitHiC or CHiCAGO [23, 71]) but it has been shown to have improved sensitivity, although the number of loops identified strongly correlates with the depth of the contact maps. A common list of loops was obtained by merging the loops lists and removing duplicates. Counts for each loop from each sample were extracted at 5 kb resolution and including the surrounding pixels, in such a way that counts were extracted from a 15-by-15-kb region around the center of the loop. These counts were extracted from SCALE normalized maps and library normalization between samples was computed by extracting the expected cis counts based on genomic distance decay and multiplying the counts by the ratio between the sample’s decay distribution with a reference sample. This distance decay function was calculated using cooltools v0.5.1 [72] api function “expected_cis” at a 10 kb resolution and calculated for each chromosome. Except for these normalization steps, no batch effect corrections were found to be necessary or influenced the results significantly.

Clustering of Hi-C libraries was performed using two separate methods. The first method is based on the loops called. Normalized counts were simply treated as data matrix and standard scaled, after which a UMAP transformation [73] was applied to the matrix to obtain the 2 component representation of the data. Alternatively, a PCA transformation could also be applied obtaining similar results. The second method was based on HiCrep [74]. We used a fast python implementation [75] which allowed the computation of pairwise distances across all samples. Distances were calculated using a bin size of 50 kb, a smoothing factor h of 5, and a maximum genomic distance of 5mb. We then applied MDS scaling on the pairwise distance matrix to generate the 2D plot in the results. Results are visualized for chromosome 1.

### RNA-seq data processing and analysis

RNA-seq reads [63] were quality filtered and trimmed and adapters were removed using fastp v0.20.1 [64]. Counts per transcript were generated using salmon v1.6.0 [76], with a decoy-aware transcriptome (Gencode hg38 v29) and the validate mappings option enabled. Counts were then aggregated per gene and differential analysis was performed using DESeq2 [77]. Genes with average counts lower than 5 were removed. In all cases, unless otherwise specified, sex was used as a covariate. For other analyses, normalized counts were extracted from DESeq2 and used. No batch effect correction was found to be necessary.

### ATAC-seq data processing and analysis

ATAC-seq reads were quality filtered and trimmed and adapters were removed using fastp v0.20.1 [64]. Reads were then mapped using bowtie2 v2.4.4 [78] using the very-sensitive pre-set. Duplicates were removed using the MarkDuplicates functionality of picard tools [79] and mitochondrial reads were removed, as well as removing low-quality alignments (MAPQ < 30) and keeping only properly paired reads. Coverages were generated from cleaned alignments using the bamCoverage functionality from DeepTools2 [80]. Peaks were called using macs2 v2.2.7.1 [81]. The ATAC-seq processing pipeline is available on GitHub and Zenodo [68, 69] (BSD 3-Clause License). Fractions of reads in peaks and fractions of reads in TSS were assayed and reported in Additional file 1: Fig. S15A–B.

Data for all samples was then analyzed in R using DiffBind [82]. A consensus peakset was generated using peaks that were present in at least 5 samples. Peak size was set to 500 bp. Cross library normalization was performed using the DESeq2 normalization method (DBA_NORM_NATIVE) but using only reads in peaks (DBA_LIBSIZE_PEAKREADS), as different samples had significantly different proportions of reads in peaks, which would bias the default normalization method. Counts were extracted at this point for further analysis, except for differential binding analysis, which was performed within DiffBind, using the DESeq2 method. No batch effect correction was found to be necessary. We found that quantile normalization slightly improved results, such as the separation of cell types on the PCA plot and correlation between expression and peak height of promoter peaks, and as such we used quantile normalized counts for all downstream analyses.

We have also used the ATAC-seq data to call genotypes for the samples for which we did not have Hi-C data. This was done using a similar method as the Hi-C data using GLIMPSE v1.1.1 [83].

### Hi-C loop differential analysis

We developed a novel method based on linear regression to perform differential analysis from a large dataset of Hi-C data. All previous tools failed to compute the large number of samples used in our experiment. We extracted and normalized counts as described in the previous sections using a distributed system across our computational cluster. For each loop, we then applied an ordinary least square regression using the python package statsmodels [84]. In the comparisons presented here, the gender of the individual was set as a co-variate. Adjusted p-values were calculated using FDR correction (Benjamin-Hochberg) as implemented in the statsmodels package. A permutation run of the data (shuffling the cell type labels) resulted in less than 3 significant differential loops.

### Differential insulation score analysis

We calculated the insulation score using the cooltools v0.5.1 [72] api function “insulation” using SCALE normalized matrixes at 25 kb resolution and a window size of 100 kb. Insulation scores were then corrected across libraries using quantile normalization. Regions with differential insulation scores were then identified using a linear regression model using the gender of the individual as a co-variate using the python package statsmodels [84]. Adjusted *p*-values were calculated using FDR correction (Benjamin-Hochberg) as implemented in the statsmodels package.

### Identification of loops and insulation bins altered in cell lines

To quantify how altered the chromatin conformation is in cell lines compared to primary cells we generated high-quality Arima Hi-C libraries for two cell lines typically used as a proxy for T cells. CD4^+^ T cells we used Jurkat E6-1 cells and for CD8^+^ T cells we used MyLa cells (Sigma-Aldrich, 95,051,033). Library generation was carried out using the same protocol as the primary cells. Sequencing was carried out to a final depth of 785 million reads for Jurkat cells and 385 million reads for MyLa cells and then processed using the same methodology as primary cells.

Loops and normalized counts were extracted as described in the previous sections. Because we had multiple replicates for the primary samples but only one replicate for the cell lines we had to test for differences in a different way. For each loop, we calculated the mean and standard deviation of this loop from our dataset and then tested if each sample was considered an outlier compared to the distribution of the counts for all samples. In practice this is calculated by calculating the *z*-score for each sample, then converting this to a *p*-value. The loop was considered an outlier if the unadjusted p-value was lower than 0.05.

Similarly, we carried out the same analysis for the insulation score. Insulation scores for all samples were pre-processed as described in the previous sections. Then for each genomic bin, we calculated the mean and standard deviation of this bin in our dataset and then tested if each sample was considered an outlier in the same way as the loops.

### Loops correlation with gene expression and chromatin accessibility

Normalized counts for each loop, gene, and ATAC-seq peak were extracted as previously described. Pearson’s correlation coefficient was calculated using the pearsonr function from scipy [85] between peak height or gene expression and interaction strength for each pairwise comparison for each sample. Because only loops that vary between the samples can show correlation, we only show the results for loops that are differentially interacting between CD4^+^ and CD8^+^ T cells (FDR < 0.01). To limit computation time, we also only show results for chromosome 1, but similar results can be shown for any other chromosome. Additionally, we only test genes that are expressed in our samples (mean count > 5), loops that have a mean interaction strength of at least 10, and peaks with at least a mean accessibility of 10 reads. The background used for peaks and genes all elements within 4 mb from the ends of the loop tested.

### Insulation score correlation with gene expression

Normalized insulation scores and counts for each gene were extracted as previously described. We then selected the genes that were differentially expressed (LFC > 1, FDR < 0.1) between CD4^+^ and CD8^+^ T cells and correlated their expression level with the insulation score of the bin which overlapped the promoter of the gene. Finally, Pearson’s correlation coefficient was calculated using the pearsonr function from scipy [85].

### CTCF tracks

Imputed CTCF tracks for primary CD8^+^ and CD4^+^ T cells were obtained from ENCODE (ENCFF148DDI and ENCFF811QTU). These have been imputed using a multi-scale deep tensor factorization method, Avocado [86, 87]. To obtain peaks, we applied a threshold to the signal of 5, corresponding to the genome-wide significance of a *p*-value of 10^−5^. Overlaps with loops and SNPs were obtained using pybedtools [88] and bedtools v2.30.0 [89].

Enrichment of GWAS variants in CTCF peaks was performed using fGWAS v0.3.6 [90].

### Identification of loops with allelic imbalance

To identify loops displaying allelic imbalance we first had to generate phased genotypes for each individual. To obtain genotypes we first mapped all Hi-C reads for each individual to the GRCh38 genome using bwa-mem v0.7.17 using the settings -SP5M. Genotypes were then called using GLIMPSE v1.1.1 [83] and the 1000 genomes phase 3 reference, making sure that all known restriction sites were excluded from the first step of the genotype calling. Genotype phasing was then carried out using the integrated phasing pipeline [91] which integrates population phasing using SHAPEIT2 [92] with reads phasing using the Hi-C using HAPCUT2 [93], resulting in around 2 million heterozygous SNPs phased per sample. We then reprocessed all Hi-C reads using Hi-C pro to a masked GRCh38 genome (all known SNPs masked with Ns) to reduce mapping bias. Aligned reads for each library were then split using SNPsplit v0.5.0 [94] generating allele-specific alignments. Counts for each allele for each significant loop (with a slop of 10 kb) were then calculated using bedtools pair_to_pair [83] and data was integrated in Python 3.9. Allelic imbalance of the reads for each loop that overlapped a SNP was tested using apeglm v1.26.0 using a beta-binomial model to account for overdispersion. Only SNP/loop pairs that had at least 3 heterozygous samples were tested. Additionally, we filtered only SNP/loop pairs with at least 10 reads present for each SNP and at least 1 read for each allele. We used calculated s-values as described in the apeglm vignette.

Allelic imbalance analysis has different trade-offs compared to QTL analysis. Please see below for further information applicable to loop allelic imbalance.

### Identification of variants with an allelic imbalance in ATAC-seq

To identify variants displaying allelic imbalance in chromatin accessibility we first reprocessed all samples by mapping them to a masked GRCh38 genome (all known SNPs masked with Ns) to reduce mapping bias. For each sample, we then identified the sites that contain variants using the bcftools v1.15.1 commands mpileup and call. Specifically, we used the following settings: mpileup -q 10 -I -E -a 'FORMAT/DP,FORMAT/AD' –ignore-RG -d 10,000, call -Aim -C alleles. Additionally, we supplied the GRCh38 fasta references as well as the 1000 genome sites, to limit the calling to known variants. Next, we filtered variants that had a read depth of at least 10 and had at least 2 reads from each allele or 5% of the reads, whichever was higher (REF and ALT). Read depth for each allele was extracted from the INFO field which contains the number of high-quality reads for the REF and the ATL alleles. Allelic imbalance for each SNP was tested using apeglm v1.26.0 using a beta-binomial model to account for overdispersion. We used calculated s-values as described in the apeglm vignette.

Overlap with caQTL was done by first selecting the caQTLs for which the variant is directly overlapping the target ATAC-peak. Then overlaps were carried out by matching the rsID of the variant. Note that whilst we can pick a corrected S-value to filter on the allelic imbalance SNP, it is harder to do so with the QTL study, as corrected p-values are calculated only on the lead SNPs. For the purposes of this overlap, we picked QTL SNPs which had an uncorrected p-value of less than 1 × 10^–4^. Finally, we compared the directionality of the allelic imbalance with the slope of the caQTL for the variants that were significant both for allelic imbalance and for caQTL. Of note, a partial explanation for the limited overlap between caQTLs and allelic imbalance SNPs is the fundamental difference in the processing of the data. In particular, we found the following limitations:

- To compare read counts for ATAC-peaks across samples, a consensus peak set needs to be generated first. This limits the extent of the size of a promoter or enhancer to 500 bp. Moreover, multiple enhancers within a certain size will be merged into one enhancer. We find that in some cases, we can split larger enhancers into multiple smaller sections, and these would correlate with genotype, whilst the overall called peak would not.
- Allelic imbalance can only be calculated if there are enough reads directly overlapping a SNP to support a heterozygous call. In some cases, reads directly overlapping the SNP can be too low to confidently call allelic imbalance, but are still sufficient for QTL analysis.
- However, allelic imbalance intrinsically corrects for batch effects and other biological variability, such as cell type composition, as the imbalance is called from within the same sample.

### Genotyping array

Genotyping was carried out using the Illumina Infinium HumanCoreExome 24 BeadChip kit (Illumina, San Diego, CA, USA). 250 ng of DNA was used, according to the manufacturer’s guidance. Genotype calling was carried out using GenomeStudio software (Illumina, San Diego, CA, USA). Standard QC was conducted on each individual array using PLINK v1.9 [95]: SNPs and samples were excluded if there was > 2% missing data, and SNPs with MAF < 0.01 and Hardy Weinberg Equilibrium (HWE) *p* < 1 × 10 − 4 were also excluded. Population stratification adjustment was done using HapMap 3 reference panel to determine the genetic ancestry of each individual, followed by Principal Component Analysis (PCA) analysis. Full genotype imputation was carried out using the HRC v1.1 reference panel on the Michigan Imputation Server [96]. Following imputation SNPs with imputation r2 score lower than 0.5 were removed.

### QTL analysis

Quantitative trait locus (QTL) analysis was performed to investigate the genetic basis of molecular phenotypes. The cis QTL analysis was carried out using QTLtools v1.3.1 [97], a versatile software that allows the use of arbitrary molecular phenotypes. We utilized genotypes called from the Hi-C data and the ATAC-seq data due to their lower error rates and fewer missing samples compared to those imputed from the genotyping array, although results were similar to the ones done using the array genotypes. Variants were filtered based on a minor allele frequency (MAF) greater than 0.05 in our samples.

For each molecular phenotype, samples that were identified as outliers through principal component analysis (PCA) were eliminated for each technique and condition. Four types of molecular phenotypes were investigated: gene expression QTL, chromatin accessibility QTL, insulation score QTL, and loop QTL.

Gene expression QTL analysis was conducted using gene expression data normalized with DESeq2 [77] and log2 transformed. Chromatin accessibility QTL analysis was performed using counts from DiffBind [82], which were subsequently quantile normalized and log2 transformed. Insulation score QTL analysis was conducted using insulation scores calculated as described previously and quantile normalized. Finally, loop QTL analysis was performed using loop counts extracted as described previously, followed by log2 transformation.

All QTL analyses were executed using the –normal parameter and tested regions within 1mb of the variant. The number of PCA components to be used as covariates for genotype and phenotype for each modality was determined by conducting a permutation pass on chromosome 1. The combination that yielded the most significant QTLs was then selected for further analysis. Disease state was tested as a covariate, but it decreased the number of significant QTLs, so it was not included in the final analysis. To calculate corrected *p*-values, we performed a permutation pass with 1000 permutations. Finally, we calculated FDR-corrected *p*-values from the adjusted p-values (fit using the beta distribution) using Benjamin-Hochberg as implemented in the statsmodels package. QQplots (expected p-value vs observed *p*-value) are shown in Additional file 1: Fig. S16.

For the overlaps between the same modality, we merged based on selecting one condition (CD4 or CD8) as the reference, which is filtered by a corrected *p*-value of 0.1. using this as the lead signal for that eQTL we then searched the other condition for the same variant-phenotype pair at an uncorrected *p*-value of 1 × 10^−2^ and reported the percentage of QTLs overlapping in this way in Additional File 3: Table S2. We then checked that the two SNPs had the same directionality in effect (slope) on the phenotype (Additional file 1: Fig. S11).

For overlaps between eQTLs and caQTLs, we matched the ATAC-seq peaks that overlapped the TSS sites for the genes in the eQTL dataset. Similarly to the previous comparison we the significant eQTLs (FDR < 0.1) and searched for caQTLs with an uncorrected *p*-value of 1 × 10^−2^. For overlaps with insQTLs, we matched the insulation score bin to the position of the TSS or the ATAC-peak. For overlaps with loopQTLs, we matched the TSS and the ATAC-seq peaks that overlapped either within the loop anchors or within the actual loop anchors, based on the description in the main text. When reporting results, we ran both CD8^+^ and CD4^+^ separately and then reported the average result between the two.

Since our dataset is relatively small compared to other publicly available eQTL datasets, we have integrated the eQTLgen dataset [15] when discussing our results, where stated in the text.

### GWAS datasets and linkage disequilibrium SNP identification

Leads SNPs for selected GWAS studies were downloaded as follows: PsA [2], Ps [98], SSc [48], atopic dermatitis [99] and RA [3], JIA [100].

SNPs in high linkage disequilibrium (*R*^2^ > 0.8) with the lead SNPs were identified using plink v1.90b3.39 on the 1000 genomes data v3 with a population set to EUR.

### *CD4* + *T-cell isolation, activation, and culture*

Primary CD4 + T cells were cultured in RPMI-1640 medium (R8758-ThermoFisher) supplemented with 10% fetal bovine serum (Gibco), 1% penicillin–streptomycin (Gibco), 200 U/ml IL-2 (Gibco) and incubated at 37 °C with 5% CO_2_. CD4 + T cells were activated using CD3/CD28 Dynabeads™ (Thermo Scientific) at a 1:1 ratio.

### Cas9 RNP knockout

CD4 + T cells were activated with Dynabeads ™ CD3/CD28 72 h prior to electroporation. Two sgRNAs were designed for the deletion, one targeting upstream (TTGGCCTCATCAGCGTGAGA AGG) and one downstream (GAGTGTCTGTATGCACCAGC AGG) of our region of interest. These gRNAs were designed using CRISPOR (found at Cispor.org). On the day, cells were electroporated using the Neon™ Transfection System 10 µL Kit (Thermo Scientific) on program #24 as recommended by the manufacturer for primary T-cells. Per reaction 300 ng of each sgRNA (Thermo Fisher) and 1250 ng of TrueCut™ Cas9 (Thermo Fisher) in 5ul Buffer T (Thermo Fisher) were added to 5 μl of CD4 + T cells (2 × 10^5^) in Buffer T. Cells were then cultured for at least 72 h after transfection before DNA was extracted using QIAamp DNA kit (Qiagen) as per manufacturer’s instructions. The site of deletion was PCR amplified (with these primers TGGCTGCATCTGACATCCTTTCGA, CCTCGCACGAAGCATGCAAACTCT) and sent to GENEWIZ for Sanger sequencing. The editing efficiency was then analyzed using the Synthego ICE tool (found at https://ice.synthego.com/#/) (Additional file 1: Fig. S17).

#### RT-qPCR

RNA was extracted from primary CD4 + T cells using the RNeasy kit (Qiagen) as per the manufacturer’s instructions. RT-qPCR was performed using the Taqman real-time PCR assays using the RNA-to-Ct 1-step kit according to the manufacturer’s instructions (Applied Biosystems). Predesigned TaqMan probes (Thermo Scientific) were used for the target gene, *BCL2L11* (Hs00708019_s1), and for the control housekeeping gene, *UBC* (Hs05002522_g1). The RT-qPCR was carried out on the Quantstudio Flex 12 K real-time PCR system (Applied Biosystems). Fold change in gene expression was subsequently calculated using the 2^−ΔΔCT^ method [101]. Fold changes were statistically analyzed using a two-tailed, unpaired, Welch’s *T*-test using GraphPad Prism version 9.0.0 for Windows, GraphPad Software, Boston, MA, USA, www.graphpad.com.

## Supplementary Information

Additional file 1. Supplementary Figs. 1–17. 

Additional file 2: Table S1. MultiQC reports and additional metadata for RNA-seq, ATAC-seq and Hi-C datasets. 

Additional file 3: Table S2. Number of significant QTLs (FDR < 0.10) that were present in the over modality at p-value 0.01 

Additional file 4: Table S3. Statistical enrichment of GWAS loci in CTCF peaks. Χ^2^ distribution with 1 degree of freedom used to calculate P values indicating the probability of observing a difference in log-likelihoods under the null hypothesis

Additional file 5: Table S4. Functional fine mapping of PsA, RA, SSc, Ps and JIA GWAS loci (online spreadsheet).

Additional file 6: Review history.

## Data Availability

All processed data, differential gene expression and chromatin accessibility analysis, QTLs, and allelic imbalance datasets are available online at: http://bartzabel.ls.manchester.ac.uk/orozcolab/SNP2Mechanism/ All the code to reproduce the results presented here is available in the same webpage, on the GitHub repository https://github.com/ChenfuShi/PsA_cleaned_analysis [68] and on Zenodo https://doi.org/10.5281/zenodo.14007665 [69]. Precomputed Hi-C correlation maps with gene expression, chromatin accessibility, and various variants are available on BioStudies (https://doi.org/10.6019/S-BSST1819) [102] and at: http://bartzabel.ls.manchester.ac.uk/orozcolab/SNP2Mechanism/PsA_output_hic_plots/main.html Processed datasets are available from GEO accession numbers GSE282511 (RNA-seq) [103], GSE282510 (Hi-C) [104], and GSE282992 (ATAC-seq) [105]. The raw data have been deposited with links to BioProject accession number PRJNA1185164 in the NCBI BioProject database (https://www.ncbi.nlm.nih.gov/bioproject/) [63]. Code deposited on Github and Zenodo is licensed under the BSD 3-Clause “New” or “Revised” License.
