# Supplementary material for: Multi-omics analysis in primary T cells elucidates mechanisms behind disease-associated genetic loci
Source: Genome Biol. 2025 Feb 10;26:26. doi: 10.1186/s13059-025-03492-y (PMC11808986; doi:10.1186/s13059-025-03492-y)
Supplement: Supplementary file 1 — Additional file 1. Supplementary Figs. 1–17. [file 13059_2025_3492_MOESM1_ESM.pdf]

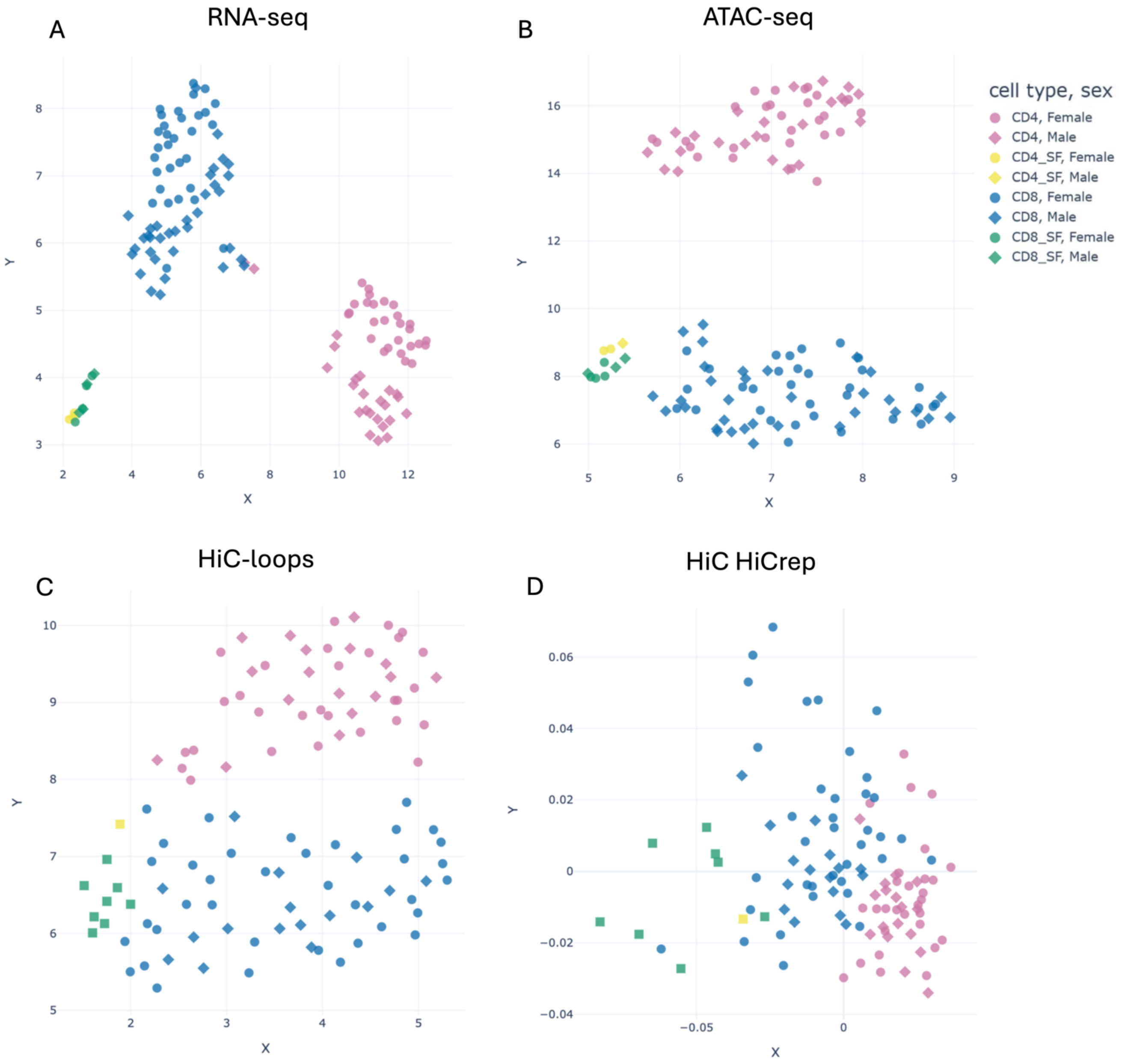

Fig S1. A) UMAP of RNA-seq data, counts from DESeq2, symbol shape is sex of individual B) UMAP of the ATAC-seq data, counts from DiffBind, symbol shape is sex of individual. C) UMAP of the Hi-C loops data, symbol shape is sex of individual. D) MDS of HiCRep analysis of the Hi-C data, symbol shape is sex of individual.

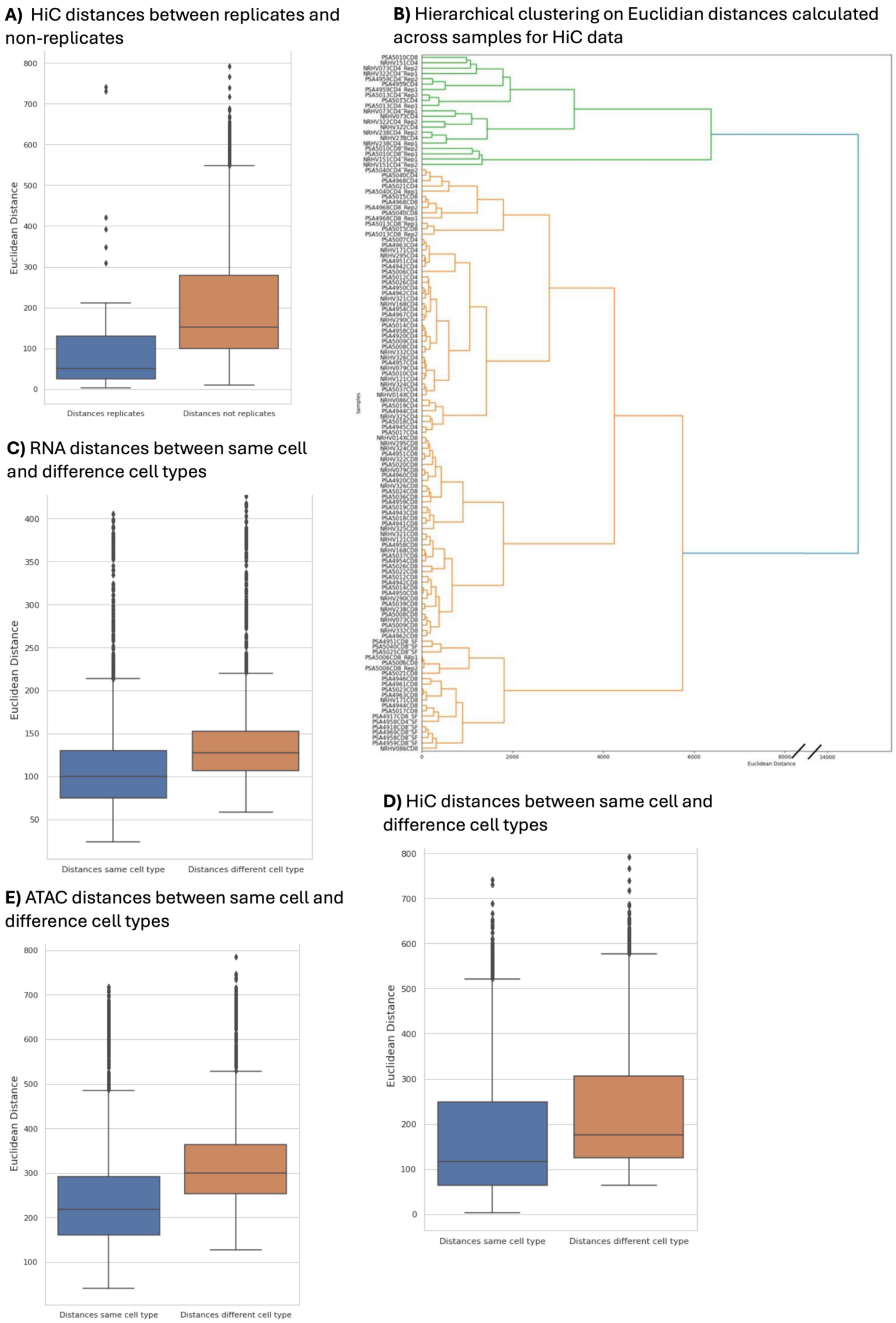

Fig S2. A) Boxplot of Euclidean distances calculated between samples coming from Replicates and non-Replicates for Hi-C data. B) Hierarchical clustering on Euclidian distances calculated across samples for Hi-C data C) Boxplot of Euclidean distances calculated between samples coming from the same cell type and different cell types for RNA-seq data. D) Boxplot of Euclidean distances calculated between samples coming from the same cell type and different cell types for Hi-C data. E) Boxplot of Euclidean distances calculated between samples coming from the same cell type and different cell types for ATAC-seq data.

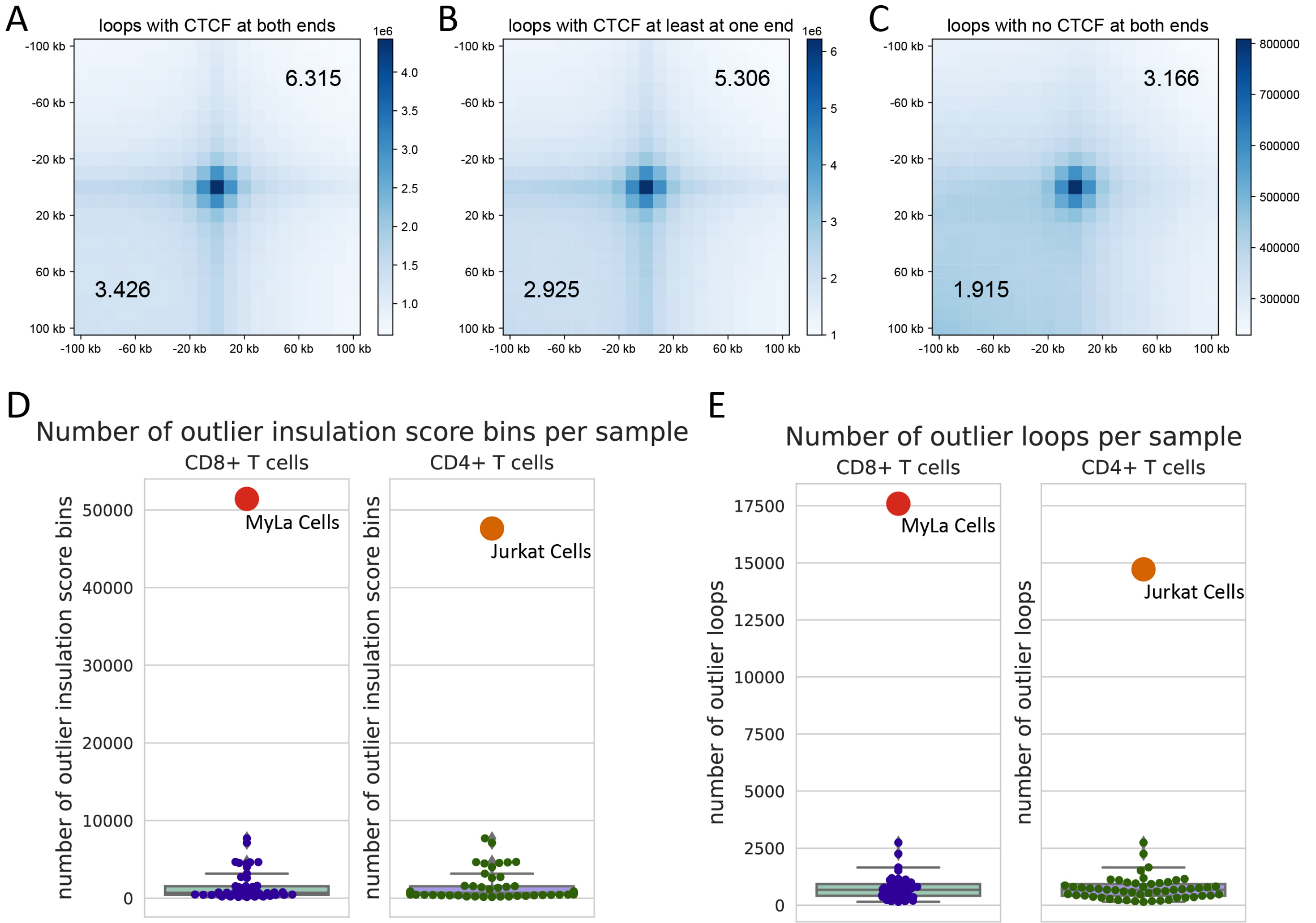

Fig S3. A) Aggregate plot analysis (APA) plot of the loops that overlap CTCF at both ends. Numbers at bottom left corner and upper right corner indicate the ratio between the central pixel and the mean of the pixels at those corners. All loops and Hi-C map are from merged CD8^{+} T cells Hi-C. B) APA plot of the loops that overlap CTCF at least at one end. C) APA plot of the loops that do not overlap CTCF. D) Boxplot with number of insulation score bins that were considered outliers, per sample. MyLa and Jurkat cells are highlighted. E) boxplot with number of loops that were considered outliers, per sample. MyLa and Jurkat cells are highlighted.

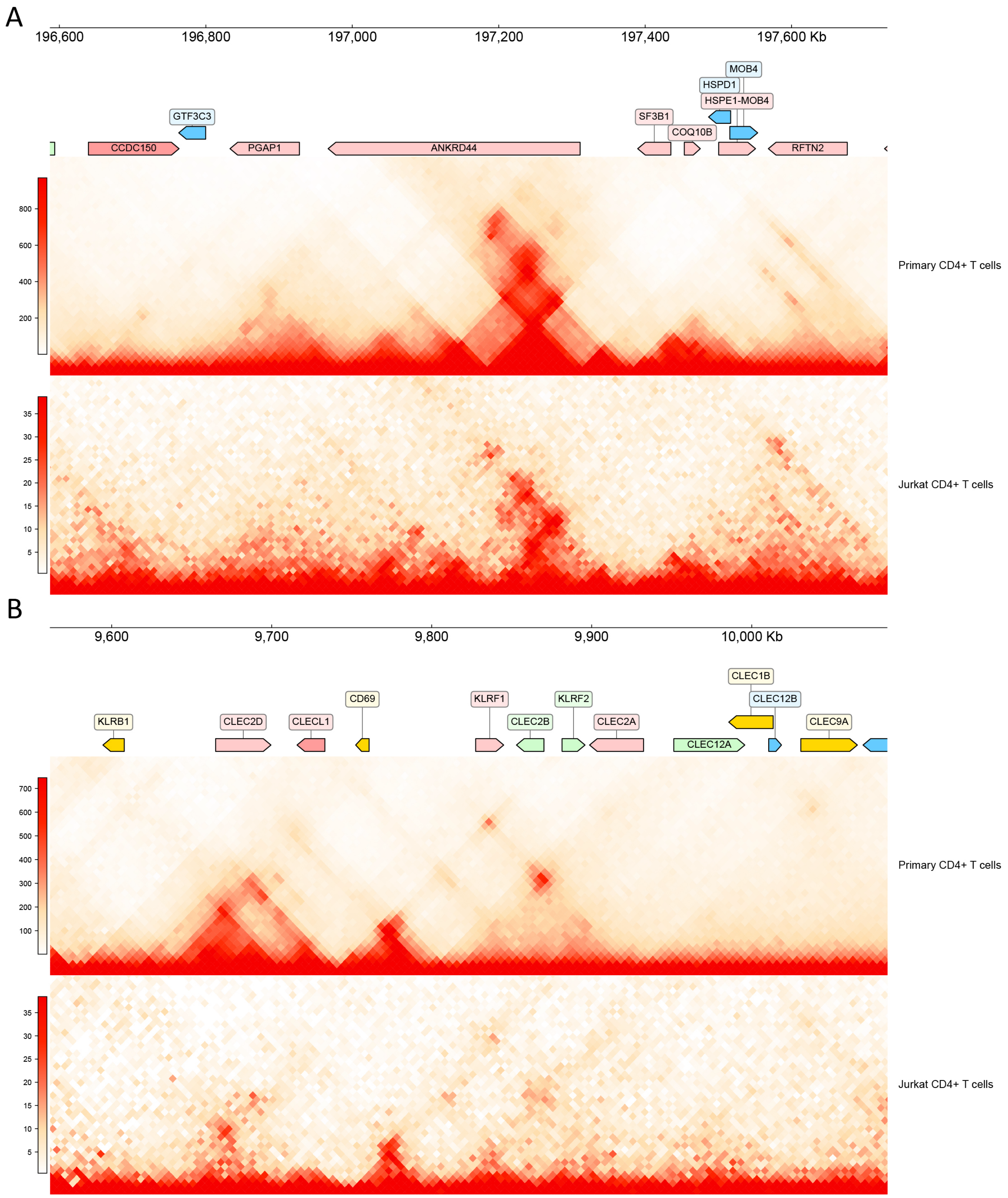

Fig S4. A) Hi-C maps from the *ANKRD44* locus, showing differences in chromatin conformation between Jurkat CD4$^+$ T cells and primary CD4$^+$ T cells. B) Hi-C maps from the *KLRF1* locus, showing differences in chromatin conformation between Jurkat CD4$^+$ T cells and primary CD4$^+$ T cells.

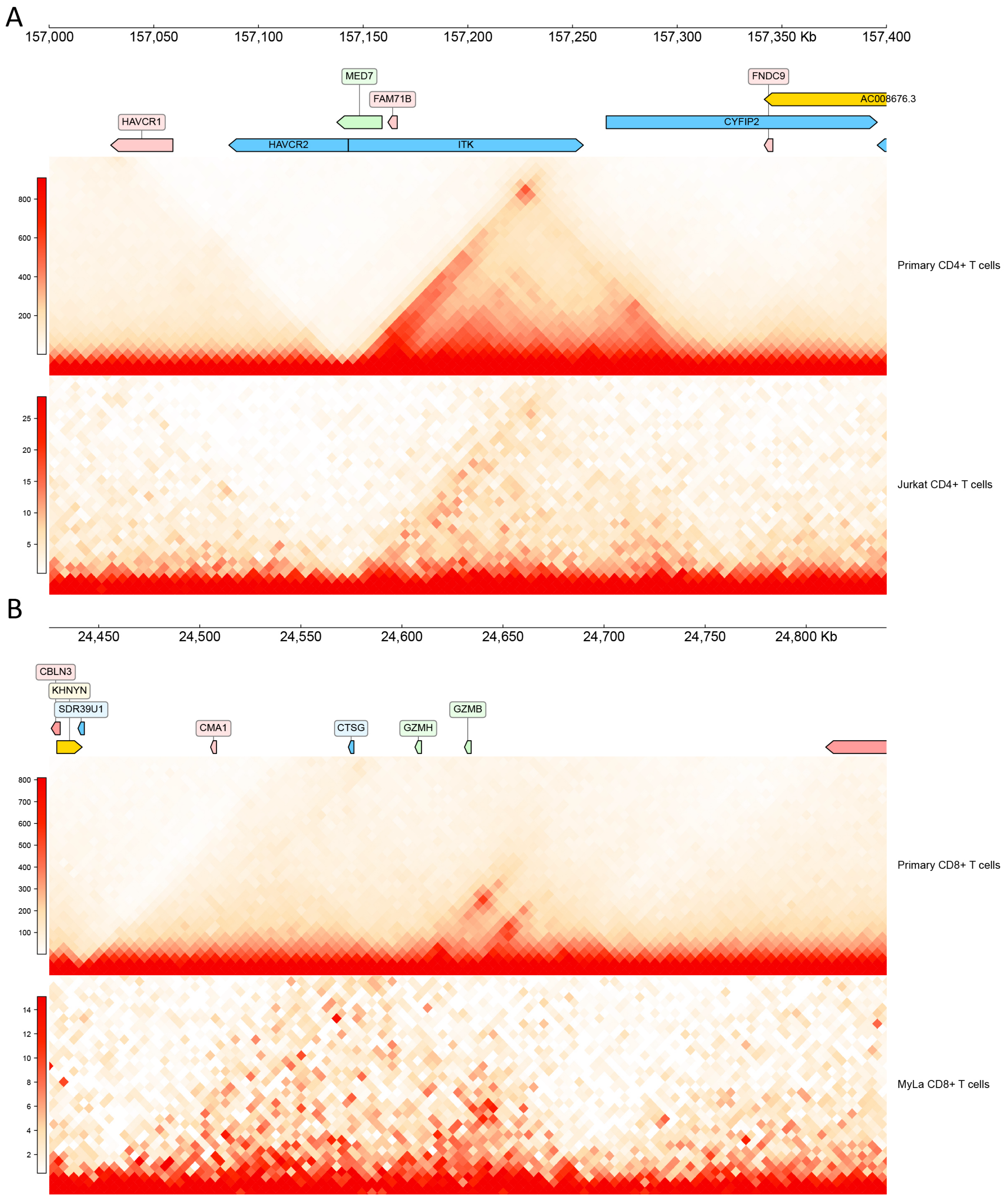

Fig S5. A) Hi-C maps from the *ITK* locus, showing differences in chromatin conformation between Jurkat CD4^{+} T cells and primary CD4^{+} T cells. B) Hi-C maps from the *GZMB* locus, showing differences in chromatin conformation between Jurkat CD4^{+} T cells and primary CD4^{+} T cells.

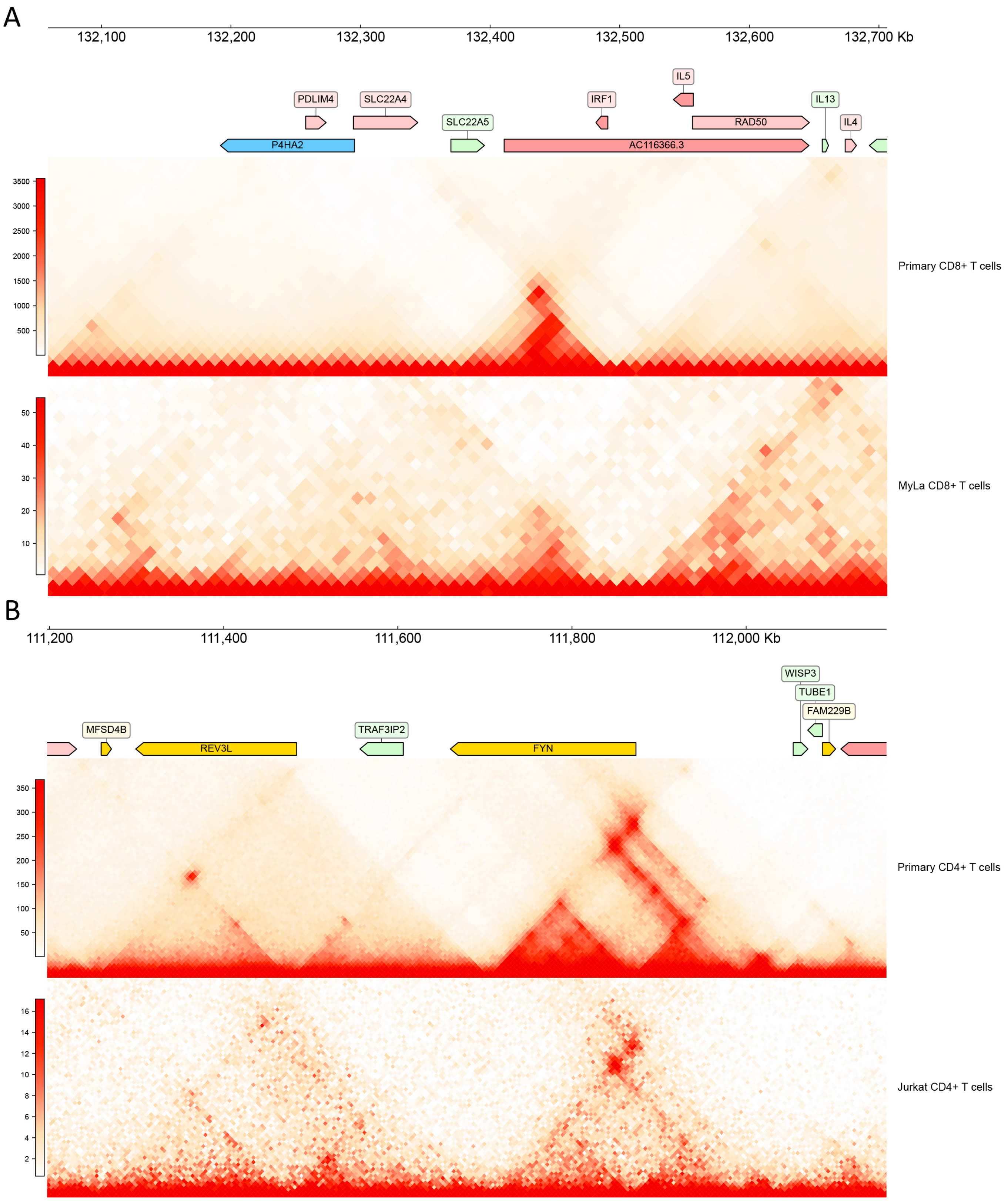

Fig S6. A) Hi-C maps from the *SLC22A5* PsA GWAS locus, showing differences in chromatin conformation between MyLa CD8$^+$ T cells and primary CD8$^+$ T cells. B) Hi-C maps from the *FYN* PsA GWAS locus, showing differences in chromatin conformation between Jurkat CD4$^+$ T cells and primary CD4$^+$ T cells.

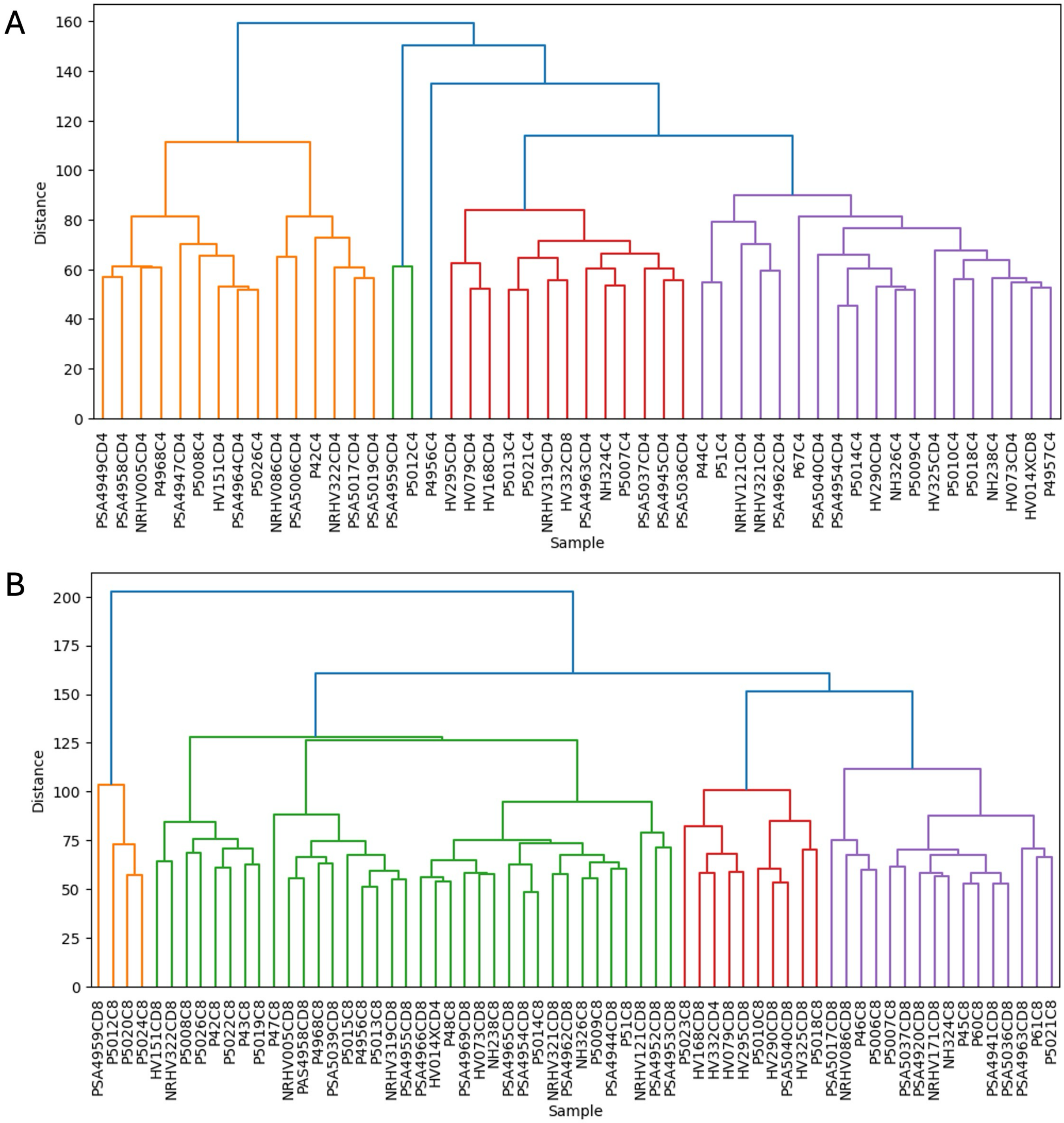

Fig S7. A) Unsupervised Hierarchical clustering of VST normalised RNA-seq count data across 18 healthy samples and 32 patient CD4 samples. B) Unsupervised Hierarchical clustering of VST normalised RNA-seq count data across 20 healthy and 46 patient CD8 samples. Each cluster is shown in a different colour.

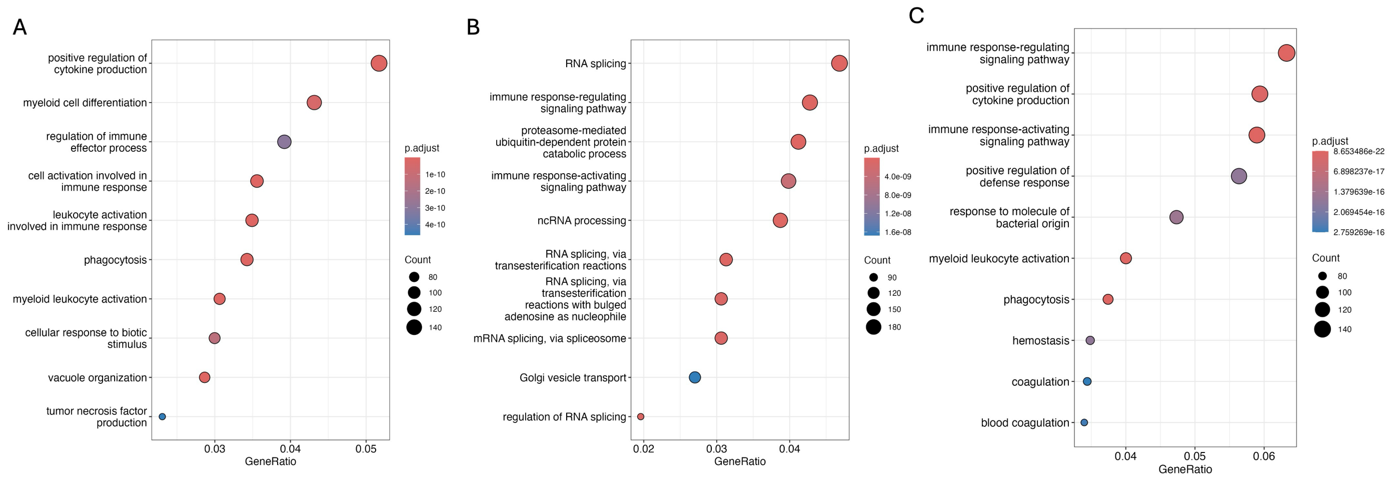

Fig S8. A) KEGG Pathway Analysis for CD4+ T Cell Samples Comparing clusters 1 vs cluster 2. B) KEGG Pathway Analysis for CD4+ T Cell Samples Comparing cluster 1 vs cluster 3. C) KEGG Pathway Analysis for CD8+ T Cell Samples Comparing cluster 1 vs cluster 2.

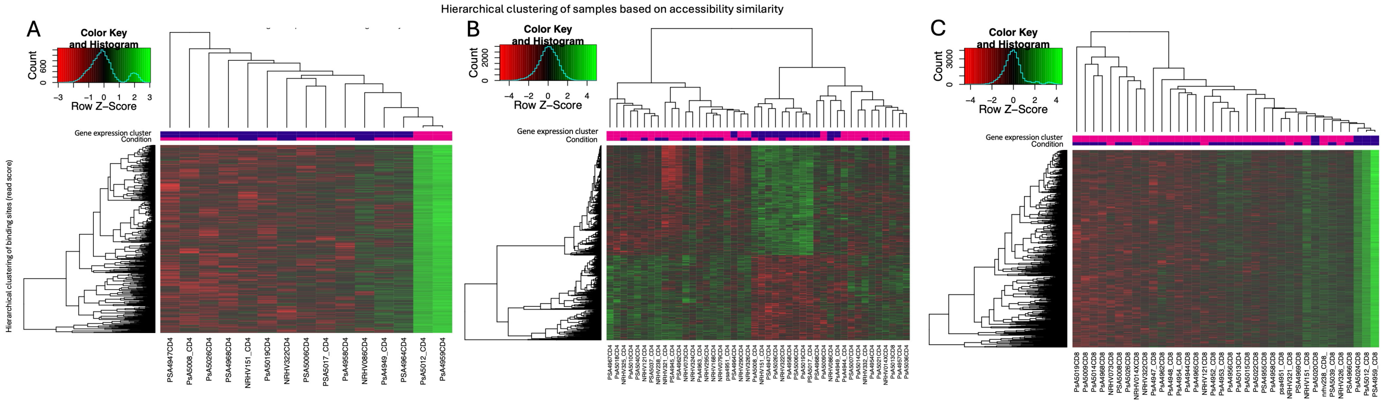

Fig S9. A) Heatmap showing differential accessibility between CD4+ samples in cluster 1 and cluster 2. Red indicates increased binding, green indicates lower binding. Blue boxes represent samples from cluster 1, pink boxes represent samples from cluster 2. B) Heatmap showing differential accessibility between CD4+ samples in cluster 1 and cluster 3. Red indicates increased accessibility, green indicates lower binding. Blue boxes represent samples from cluster 1, pink boxes represent samples from cluster 3. C) Heatmap showing differential accessibility between CD8+ samples in cluster 1 and cluster 2. Red indicates increased accessibility, green indicates lower binding. Blue boxes represent samples from cluster 1, pink boxes represent samples from cluster 2.

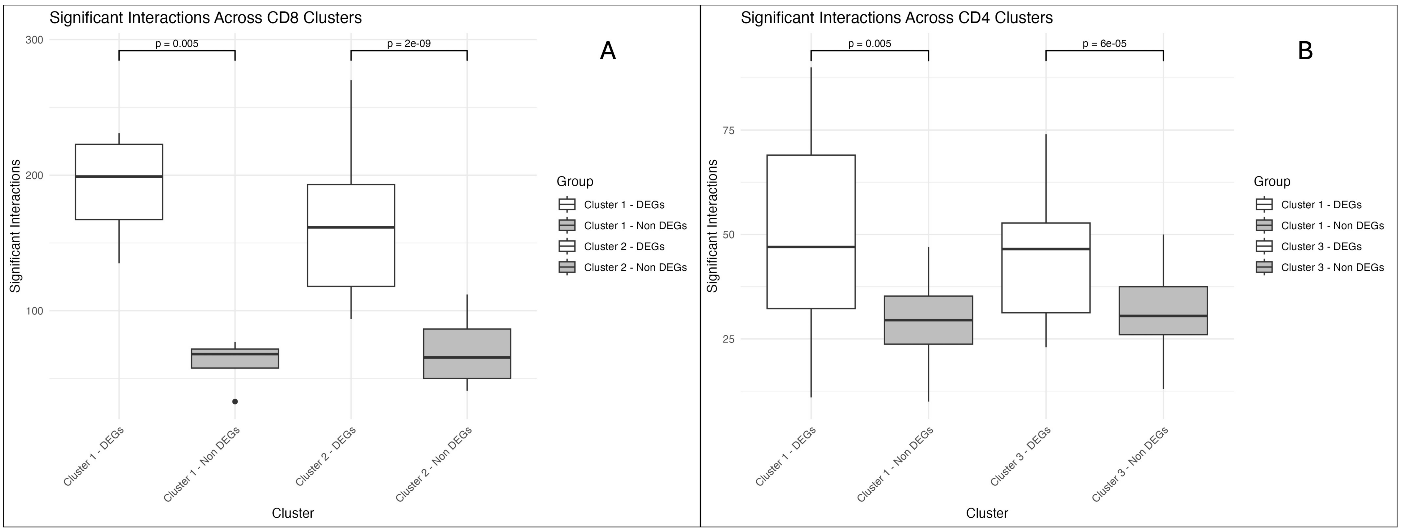

Fig S10. Distribution of significant interactions between cluster specific differentially accessible peaks and differentially expressed genes/non-differentially expressed genes in CD8+ T-cells (A) and CD4+ T-cells (B).

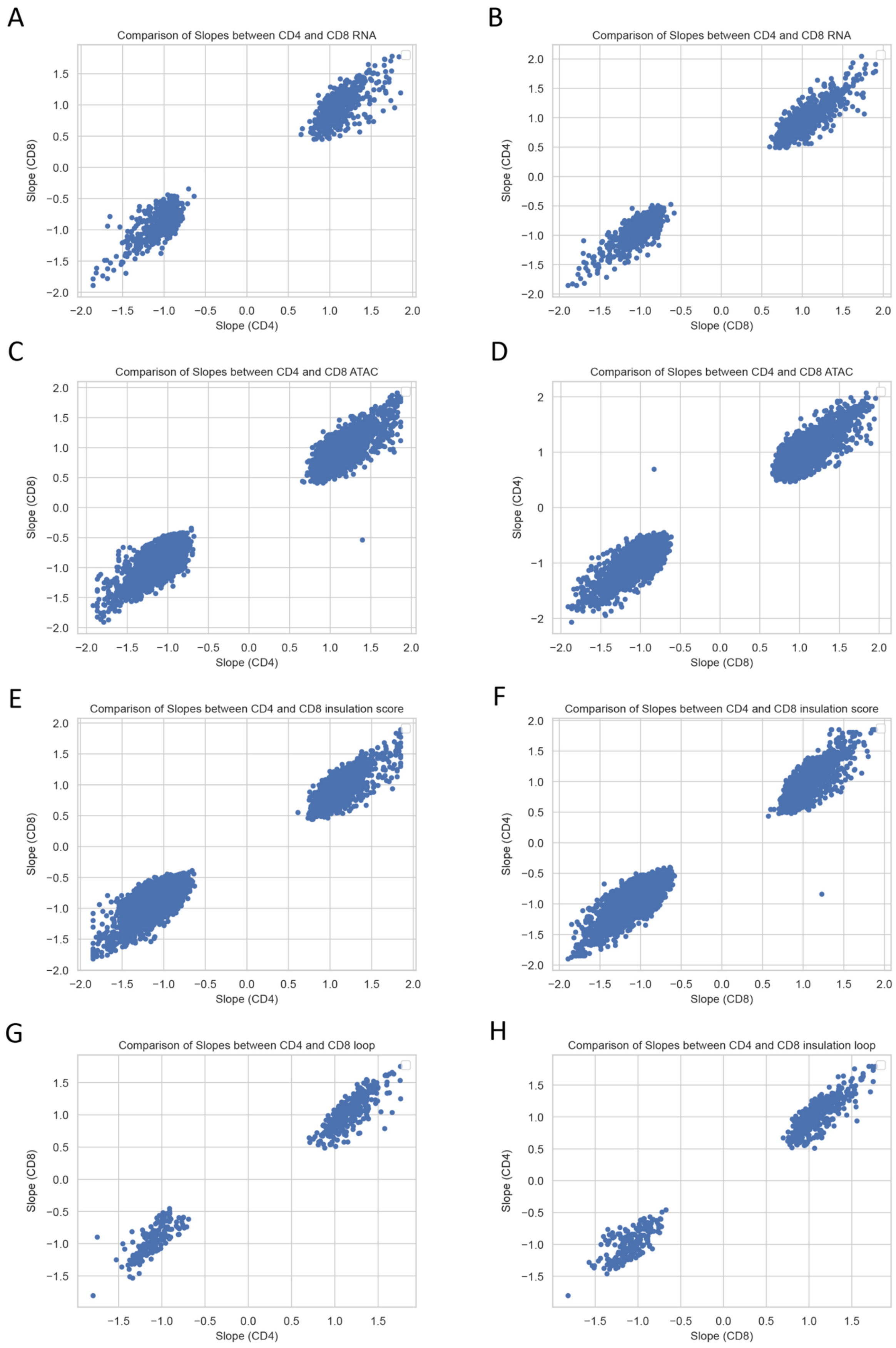

Fig S11. A) Correlation of slopes between significant (FDR<0.1) eQTLs called in $CD4^+$ and their counterpart (p-value < 0.01) in $CD8^+$ T cells. B) Correlation of slopes between significant (FDR<0.1) eQTLs called in $CD8^+$ and their counterpart (p-value < 0.01) in $CD4^+$ T cells. C) Correlation of slopes between significant (FDR<0.1) caQTLs called in $CD4^+$ and their counterpart (p-value < 0.01) in $CD8^+$ T cells. D) Correlation of slopes between significant (FDR<0.1) caQTLs called in $CD8^+$ and their counterpart (p-value < 0.01) in $CD4^+$ T cells. E) Correlation of slopes between significant (FDR<0.1) insQTLs called in $CD4^+$ and their counterpart (p-value < 0.01) in $CD8^+$ T cells. F) Correlation of slopes between significant (FDR<0.1) insQTLs called in $CD8^+$ and their counterpart (p-value < 0.01) in $CD4^+$ T cells. G) Correlation of slopes between significant (FDR<0.1) loopQTLs called in $CD4^+$ and their counterpart (p-value < 0.01) in $CD8^+$ T cells. H) Correlation of slopes between significant (FDR<0.1) loopQTLs called in $CD8^+$ and their counterpart (p-value < 0.01) in $CD4^+$ T cells.

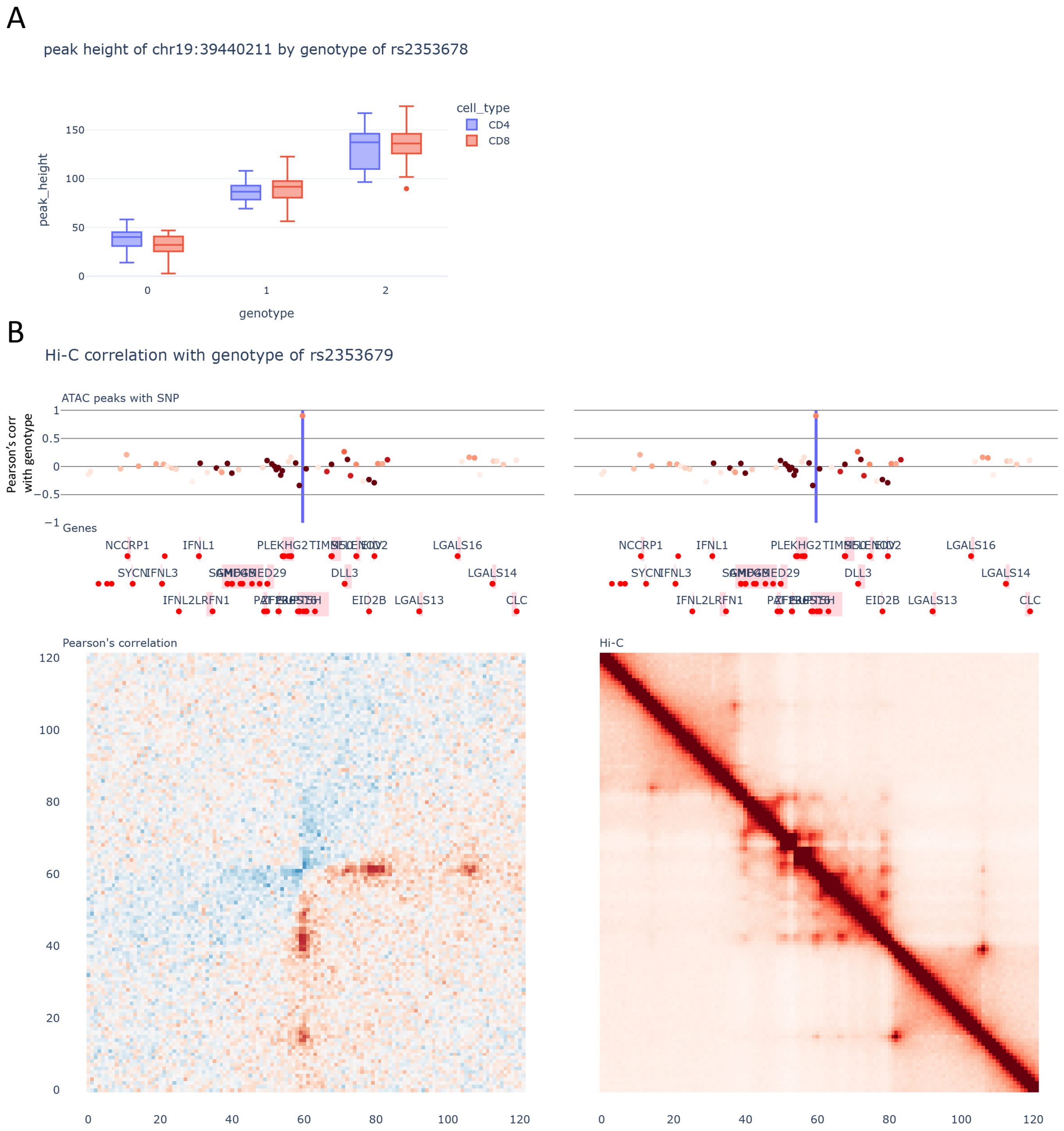

Fig S12. A) Accessibility of CTCF site located at chr19:39440211 vs the genotype of rs2353678. B) Visualization of the region surrounding rs2353678. Top: ATAC-seq peaks. Intensity of the colour indicates the average peak-height of the peak, whilst position across the Y axis indicates the pearson's correlation with the genotype of rs2353678. Middle: Genes from Gencode v29. The red dots indicate the transcription start sites. Bottom left: Correlation between the Hi-C contacts and the genotype of rs2353678. Bottom right: Merged Hi-C map.

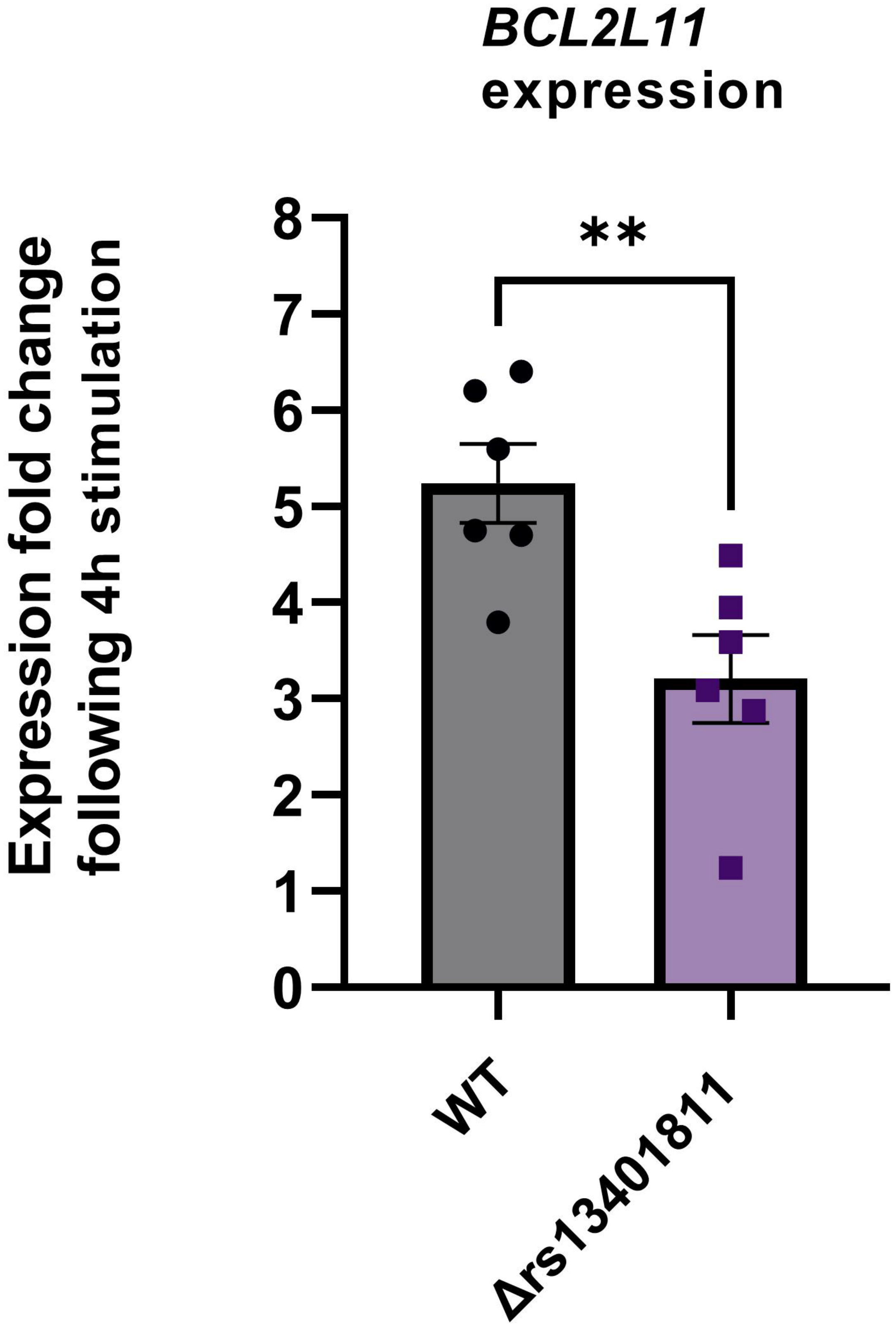

Fig S13. The rs13401811 enhancer region regulates *BCL2L11* expression in primary human CD4+ T cells. Following a 4-hour stimulation with CD3/CD28 beads RT-qPCR showed *BCL2L11* expression increased 5.2-fold in WT cells and 3.2-fold in Δrs13401811 cells (P = 0.008, n = 6). Error bars represent standard error of the mean (SEM). Statistical significance was determined using a two-tailed, unpaired, Welch's T-test (**p < 0.01).

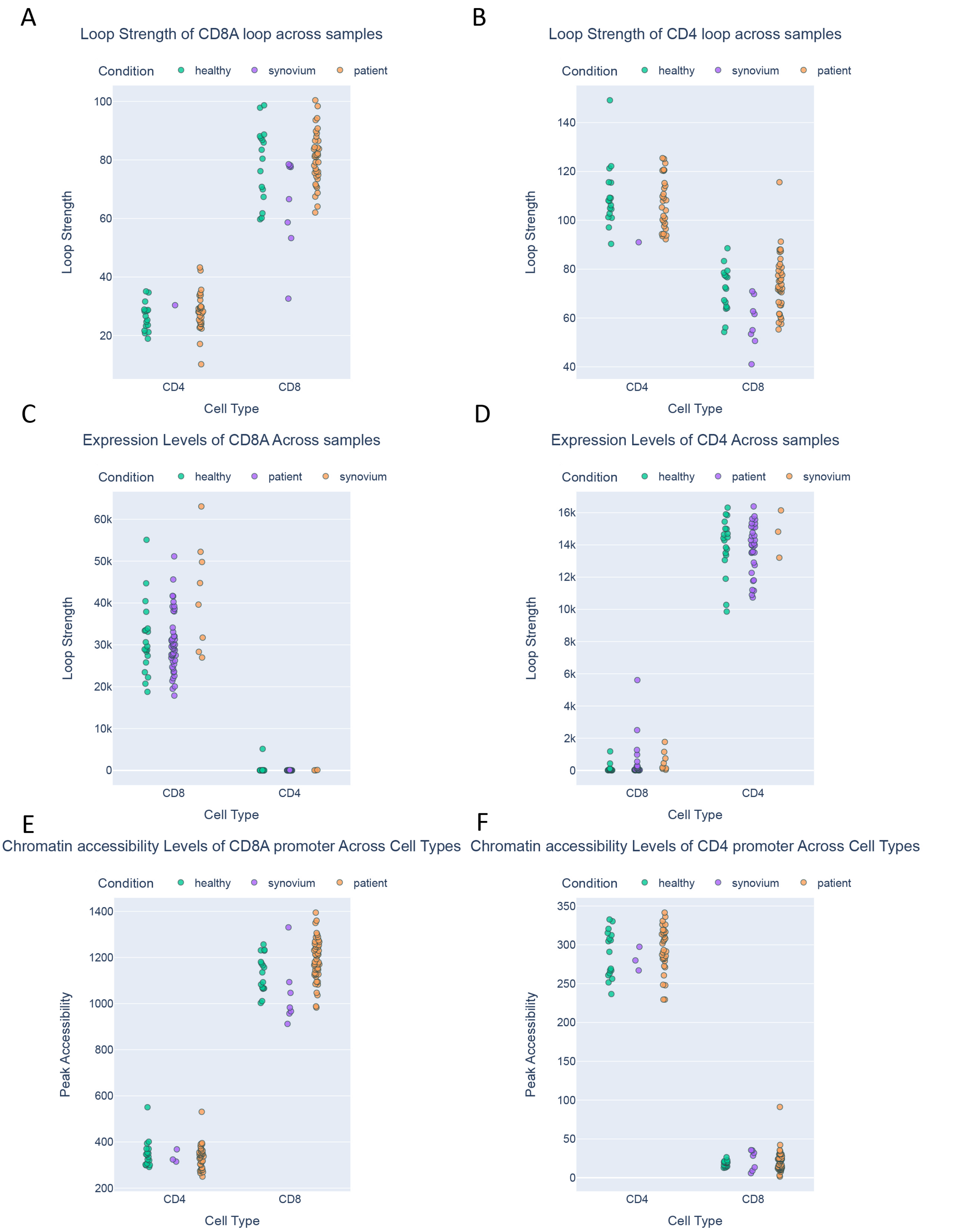

Fig S14. A) Loop strength levels of loop localised around *CD8A* promoter across samples. B) Loop strength levels of loop localised around *CD4* promoter across samples. C) RNA-seq expression levels of *CD8A* across samples. D) RNA-seq expression levels of *CD4* across samples. E) ATAC-seq chromatin accessibility levels of *CD8A* promoter across samples. F) ATAC-seq chromatin accessibility levels of *CD4* promoter across samples.

A

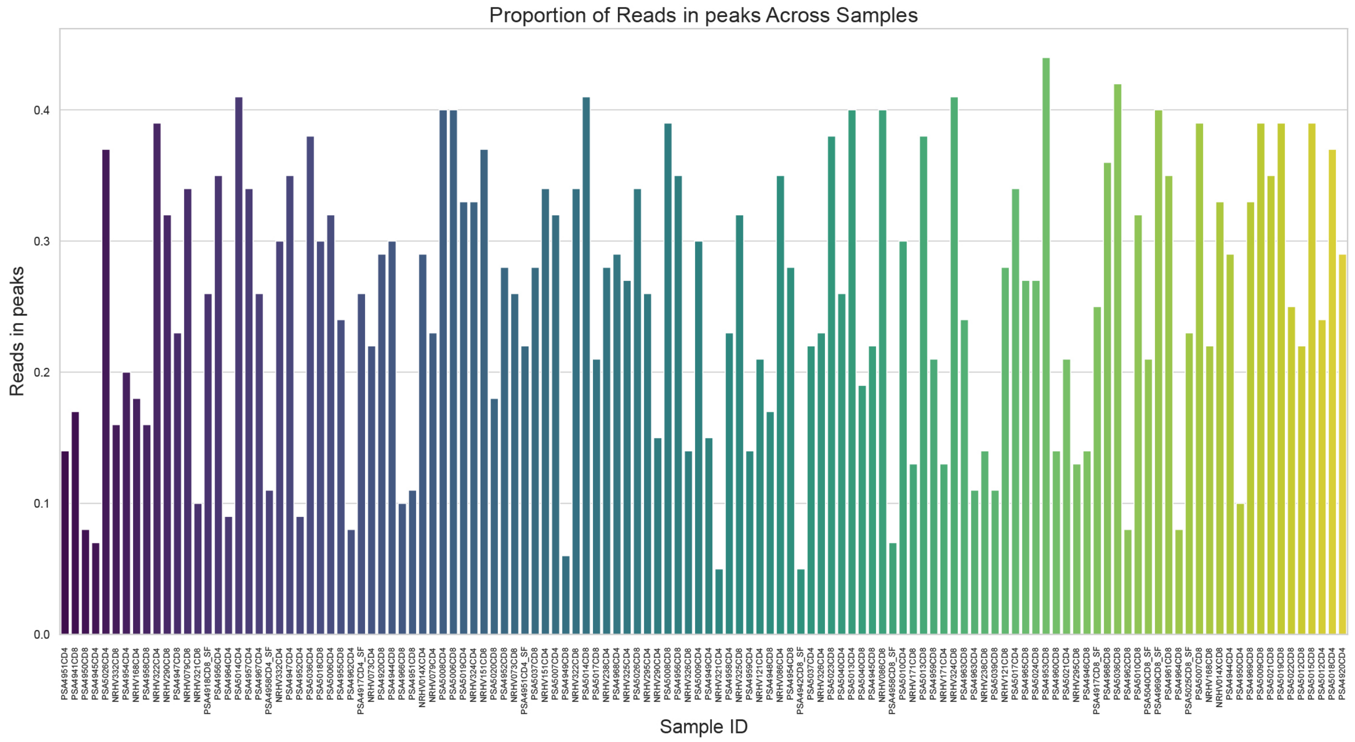

B

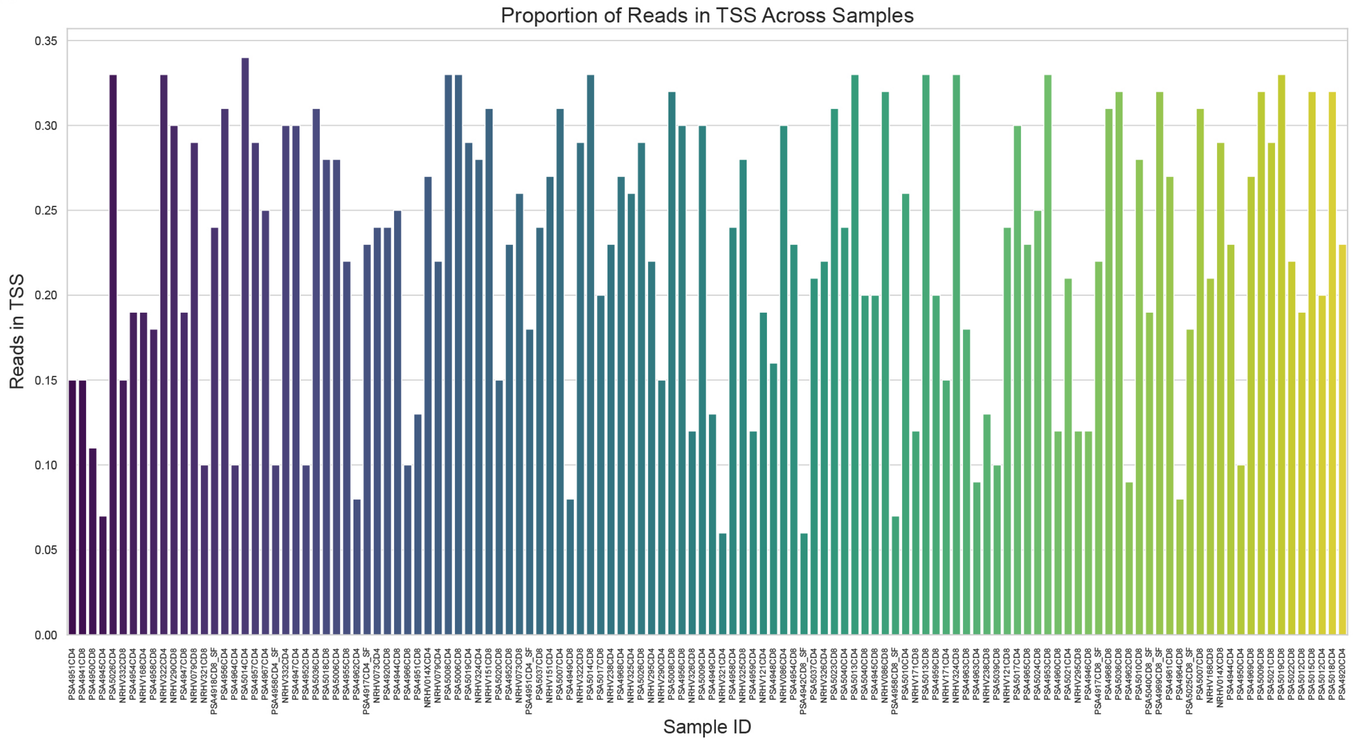

Fig S15. A) Fraction of reads in peaks (FRiP) for all ATAC-seq samples. B) Fraction of reads in TSS (FRiTSS) for all ATAC-seq samples.

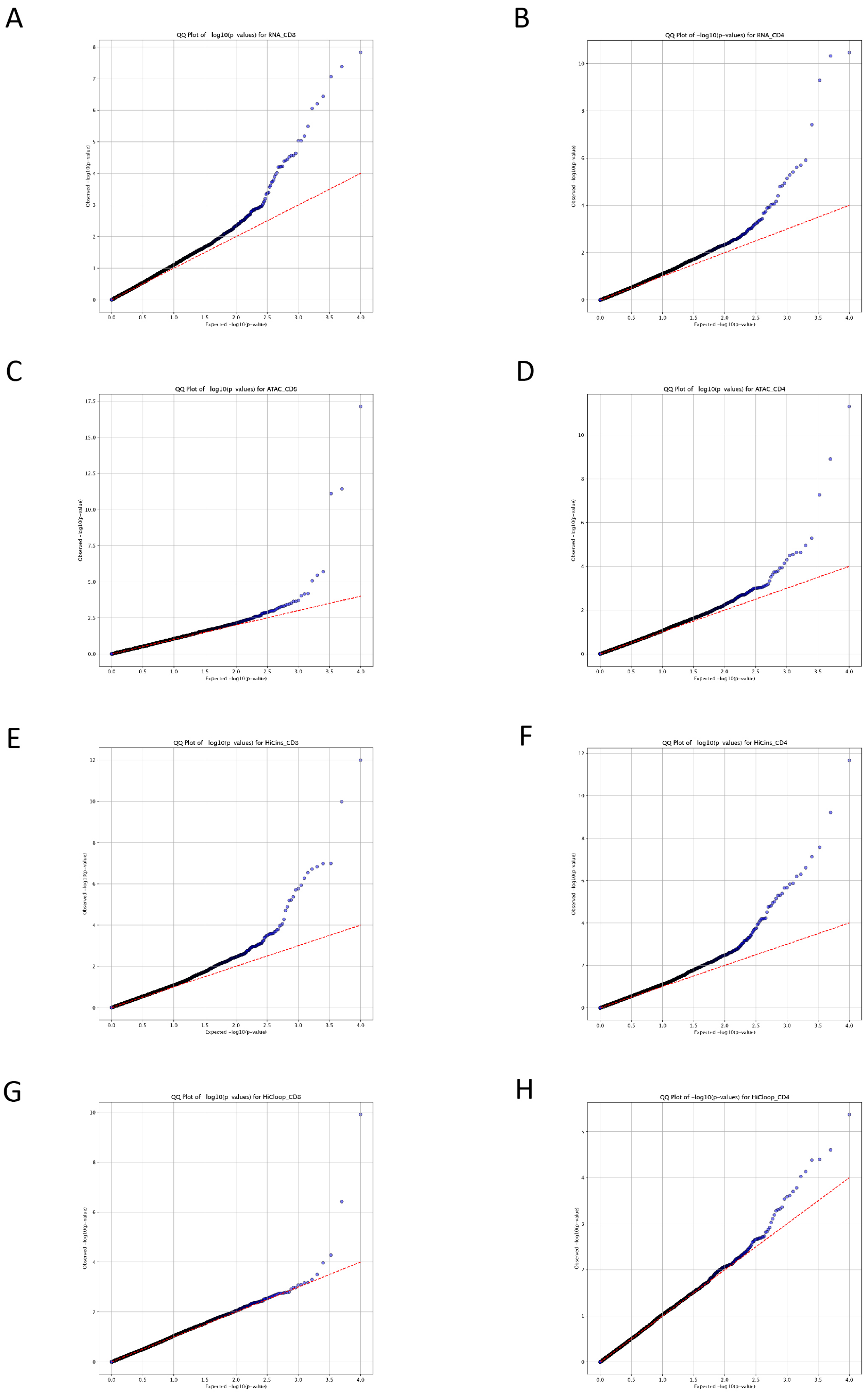

Fig S16. A) QQ plots of expected p-values vs observed p-values for eQTLs in CD8⁺ T cells. A) QQ plots of expected p-values vs observed p-values for eQTLs in CD4⁺ T cells. C) QQ plots of expected p-values vs observed p-values for caQTLs in CD8⁺ T cells. D) QQ plots of expected p-values vs observed p-values for caQTLs in CD4⁺ T cells. E) QQ plots of expected p-values vs observed p-values for insQTLs in CD8⁺ T cells. F) QQ plots of expected p-values vs observed p-values for insQTLs in CD4⁺ T cells. G) QQ plots of expected p-values vs observed p-values for loopQTLs in CD8⁺ T cells. H) QQ plots of expected p-values vs observed p-values for loopQTLs in CD4⁺ T cells.

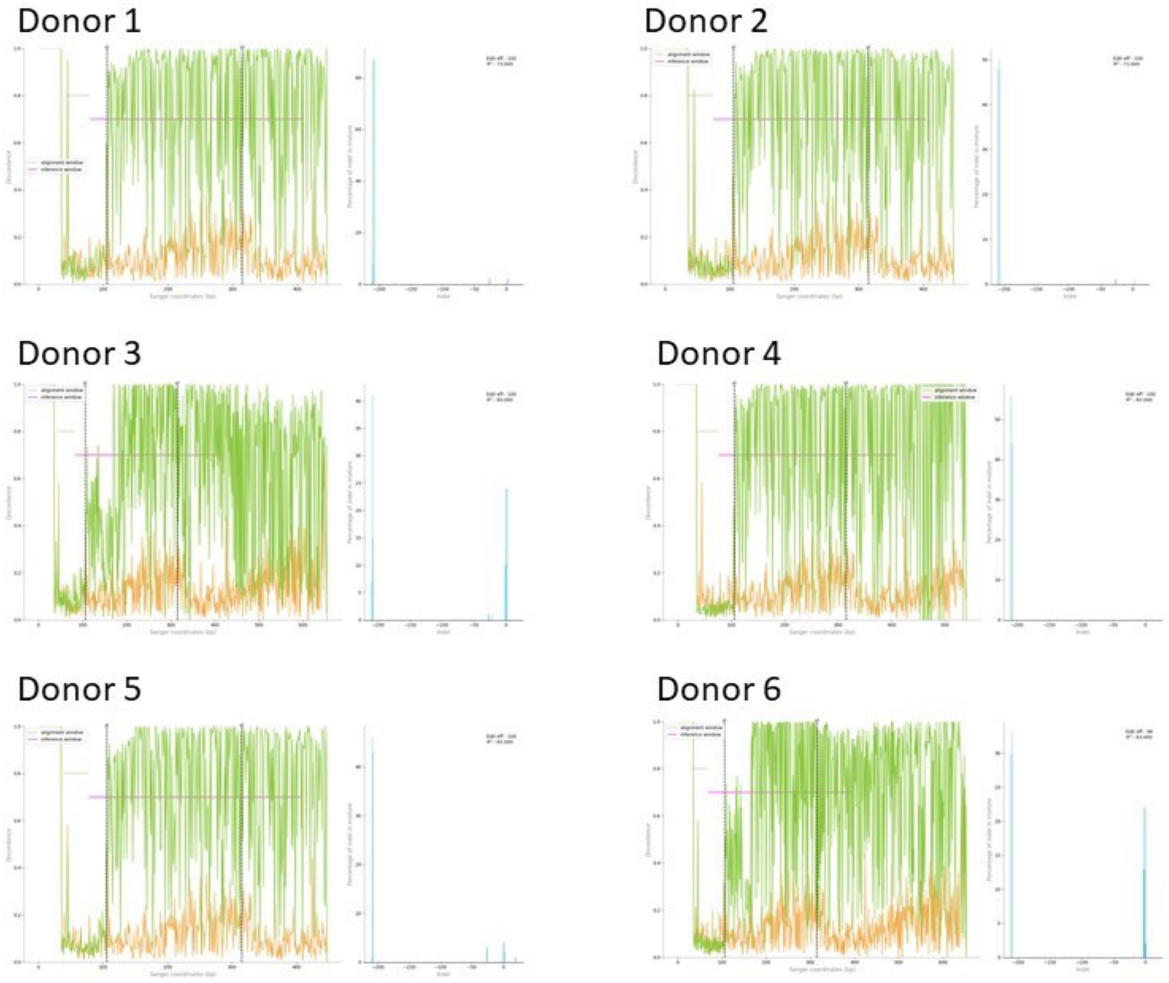

Fig S17. *BCL2L11* KO ICE analysis. Analysis of Sanger sequencing chromatograms of the rs13401811 target region from unedited and edited (Δrs13401811) CD4+ T cells from 6 donors. ICE analysis results the distribution of deletion sizes and their respective frequencies, highlighting a predominant ~200bp deletion.
