# Supplementary material for: Multi-omics analysis in primary T cells elucidates mechanisms behind disease-associated genetic loci
Source: Genome Biol. 2025 Feb 10;26:26. doi: 10.1186/s13059-025-03492-y (PMC11808986; doi:10.1186/s13059-025-03492-y)
Supplement: Supplementary file 6 — Additional file 6: Review history. [file 13059_2025_3492_MOESM6_ESM.docx]

Review History

**First round of review**

**Reviewer 1**

**Were you able to assess all statistics in the manuscript, including the appropriateness of statistical tests used?**

No, I do not feel adequately qualified to assess the statistics.

**Comments to author:**

This study investigates the interplay among gene expression, chromatin accessibility, and 3D genome organization in CD4+ and CD8+ primary T cells from patients with psoriatic arthritis (PsA) and healthy controls. The authors have generated a vast amount of data, including 108 Hi-C libraries with 49B read depths, to link genetic variants to the variability of gene expression, chromatin accessibility, and 3D genome organization. However, despite the valuable resources provided, the authors fail to provide a well-confirmed conclusion. As acknowledged by the authors themselves, the variability originating from sample heterogeneity, experimental variations, and analysis limitations outweighs the biological variability targeted in this study. Thus, the authors should reposition this valuable resource to reach more robust conclusions. Below are more specific comments:

1. The figure resolution is too low to interpret the results. The authors should provide high-resolution images.
2. Detailed clinical information about the patients should be provided to enhance the understanding of the results. The clinical information for the samples collected in this study was not accessible from the link provided by the authors.
3. The authors should confirm the expression levels of marker genes in both patients and controls for each cell type. Also, the authors should examine the variability of the maker gene expression for each cell type and investigate whether such variation indicates the presence of potential unknown confounding factors.
4. The authors should provide results on the reproducibility of the findings for selected samples. It is challenging to determine whether the heterogeneity issue in the multi-omics results reflects sampling heterogeneity, patient heterogeneity, or individual heterogeneity. At least the authors should collect samples twice from the same patients and determine the degree of reproducibility.
5. The authors essentially stratified the analysis in terms of active vs. non-active disease and active disease vs. controls. However, given the identified limited number of differentially expressed genes, the authors should clarify why such a comparison is biologically meaningful.
6. To demonstrate their results clearly, the authors need to perform clustering analysis of gene expression data and identify subpopulations based on molecular phenotypes (i.e., gene expression). Subsequently, for each group, the authors need to integrate chromatin accessibility and Hi-C results to explain the variation in gene expression patterns within each group. Without such clear stratified analyses for each omics result, it is hard to justify the rationale for the integration of multi-omics results.
7. The authors may have reached overly conclusive statements due to limited data analysis. For example, the authors concluded that 'the way genetic variants affect these interactions is not primarily driven by alteration of the CTCF binding.' However, without CTCF ChIP-seq data for each individual, it is difficult to determine whether alteration of CTCF binding is a factor driving 3D genome variability.
8. The authors suggest several GWAS loci and how the variants potentially involve pathogenic gene regulation in the context of chromatin accessibility and chromatin loops, but without minimum experimental validation using genome editing, it is challenging to confirm such conclusions. The authors should present at least one experimentally validated example linking GWAS variants to molecular phenotypes.

**Reviewer 2**

**Were you able to assess all statistics in the manuscript, including the appropriateness of statistical tests used?**

Yes, and I have assessed the statistics in my report.

**Comments to author:**

The authors of this paper have generated a nice dataset of Hi-C, ATAC-seq and RNA-seq from primary CD4+ and CD8+ T cells isolated from peripheral blood and synovial fluid of psoriatic arthritis patients and normal individuals. The Hi-C data in particular are a unique resource, that is far larger than prior Hi-C studies in primary cells. The dataset is a major strength of the paper. The Hi-C data allows the authors to detect the effects of gene expression and chromatin QTLs are chromatin architecture at a high resolution. There are some good examples of this shown in the paper, such as at the IKZF3/GSDMB/ORMDL3 locus. There are several weaknesses in the paper. A few more QC steps should be taken. The QTL analyses have a several issues, including possibly incorrectly applied thresholds for statistical significance and multiple testing correction. These issues are described in detail below.


Major Comments:
1) One RNA-seq sample is likely to be mislabeled as CD4 patient that is actually CD8, based on UMAP shown in Fig 1B. Suggest checking expression of CD4/CD8 specific marker genes and reassigning sample label if necessary.
--

2) Related to the above point, I suggest checking RNA-seq/Hi-C/ATAC-seq samples are concordant with genotyping from SNPChip. In most large studies like this one, there are a few sample mixups where the genotypes don't match the assays. These can be resolved by looking at pairwise correlations between assay genotypes. One tool to do this is NGSCheckMate.
--

3) Additional QC of the ATAC-seq data should be performed. ATAC-seq quality can vary dramatically, so it is important to calculate the fraction of reads in peaks (FRiP) and TSS enrichment score for each sample. Samples that do not meet QC thresholds should likely be removed from the study. ENCODE has some guidelines on this (https://www.encodeproject.org/atac-seq/) although their minimum thresholds may be too strict for primary cells, which generally give lower quality data than cell lines.
--

4) Table 2 describes total number of insulation score QTLs (insQTL), loop strength QTLs (loopQTL), gene expression QTLs (eQTLs) and chromatin accessibility QTLs (caQTLs). It's not clear what the sample sizes are, and I would suggest adding this to the table. I would guess that the sample sizes are all 74 (n=55 PsA patients + n=19 healthy controls) but it's not obvious that this is the case or if the numbers are consistent across assays/cell types.
--

5) The numbers of reported significant QTLs seems too high given sample sizes. The number of eGenes seems on the high side (see Fig 2A in the GTEx paper for comparison https://pubmed.ncbi.nlm.nih.gov/32913098/). Based on my experience with ATAC-seq datasets of size n=100-150, the number of caQTLs identified seems much too high. I would expect the number of caQTLs identified to be about 10,000 or lower with this dataset (~5% of total peaks), rather than the reported 19-26k.
--

6) Because of the point above, and the methods section, I am concerned that an inappropriate p-value threshold has been used to determine significance. The methods say: "To calculate corrected p-values, we performed a permutation pass with 1000 permutations. However, corrected p-values are only calculated on the top hit. We find that for all our modalities an equivalent uncorrected pvalue to a corrected p-value of 0.1 is approximately 1x10-4. For the calculation of the overlaps between the modalities, we decided to use this latter p-value as the top hit would rarely overlap between the different modalities."

Based on the above description, there appears to be some confusion about the nature of the p-values returned from QTLTools and how they should be used. In QTL analysis, there is a multiple testing problem for each gene (or peak or loop) considered because many SNPs are tested for association with each gene (or peak). Because the tested SNPs are not independent (due to LD) a standard FDR correction may not be appropriate. A per-locus p-value correction is typically done by performing permutations that maintain the correlations between nearby SNPs (LD). Adjusted p-values are then computed from permutations using the most significant p-value from each permutation, as described in the FastQTL paper. The adjusted p-value should be interpreted as a p-value for the gene (i.e. encompassing all of the SNPs that are tested). The statement that "the corrected p-values are only calculated on the top hit" is not totally correct—even though the top hit is used to estimate the corrected p-value in the permutations, the permutation p-value should not be viewed as assigned to a particular SNP--rather it is for the entire locus. In other words, a low corrected p-value means that there is at least one significant association between genotypes of the SNPs at the locus and the expression of the gene. You can use the adjusted p-value to identify significant eGenes, and then use the most significant SNP as the lead SNP. (side note: if you want to identify more than one association per gene, then a conditional analysis needs to be performed (QTLTools has the ability to do this)).

The authors use a nominal p-value cutoff of 1e-4, which is claimed to be roughly equivalent to an adjusted p-value of 0.1 (it's also not appropriate to use an adjusted p-value of 0.1). First, I doubt that this approximation will hold for all loci, because the relationship between nominal p-values and the adjusted p-value depends on the number of SNPs tested at the locus and on LD. Second, while the adjusted p-value accounts for the number of SNPs tested for a given gene (or peak or loop) I don't think it accounts for the total number of genes tested (or peaks or loops). This becomes a larger problem when more elements are tested, such as in the case of peaks and loops .

I suggest that the authors should take the adjusted p-values output by QTLTools (the ones computed using the Beta distribution), and then compute an FDR across all tested genes (or peaks or loops) using the adjusted p-values to determine which genes have significant eQTLs. The same approach should be applied to the other QTL types. I also recommend that the authors make qqplots of -log10 observed vs. expected p-values so that the calibration of the p-values can be examined.
--

7) The low concordance of overlaps of loopQTLs between CD4 and CD8 T cells is concerning, and suggests that potentially most of the identified loopQTLs are false positives. This may relate to the use of inappropriate p-value thresholds discussed above rather than sensitivity as hypothesized by the authors. It would be good to see how overlap changes as a function of significance.

8) The overlaps analysis for eQTLs and caQTLs doesn't seem correct. The methods state "For overlaps between eQTLs and caQTLs, we matched the ATAC-seq peaks that overlapped the TSS sites for the genes in the eQTL dataset." Why should only peaks overlapping the TSS be used? Shouldn't it be possible to consider distal caQTLs for overlap with distal eQTLs?

9) The allelic imbalance analysis for both chromatin accessibility and chromatin loops uses a binomial test. The binomial test for allelic imbalance is known to give a lot of false positives due to overdispersion in genomic count data, and for this reason a beta-binomial test is generally used instead. Also, a two-sided test should be used, not a one-sided test.

10) The analysis of the overlap between variants and CTCF binding sites is difficult to interpret without some information about the null expectation. For example, for the statement "15% of loops with allelic imbalance have one variant located within a CTCF peak", is this an enrichment over what is expected by chance?


Minor Comments:

11) I strongly suggest putting more information about the number of samples, type of samples and cell types in the abstract of the paper. The dataset is a really important aspect of the paper, but currently it is not described until the results section.
"chromatin accessibility and gene expression from freshly isolated primary CD4+ and CD8+ T cells from 55 PsA patients and 19 Healthy controls (Figure 1A). This consists of 108 Hi-C libraries (49 billion reads), 128 RNA-seq libraries and 126 ATAC-seq libraries"

12) "with the synovial fluid samples forming a separate cluster from the peripheral blood samples" Suggest mentioning that T cells were obtained from peripheral blood and synovial fluid prior to this sentence.

13) P6, L33: following reference to "Supplementary Figure 5" there is an error message in the PDF:: "Error! Reference source not found"

14) P6, L40-42: Differences between CD8+ blood / synovial are described but what about CD4+ cells? They seem left out here.

15) Fig 1F: shows correlation between gene expression and insulation score. Legend states that each dot is a gene, but then there are a fairly small number of genes included in this plot. The legend should state which subset genes are used here, since doesn't appear to be all genes.

16) P7: Throughout the paragraph on correlations between chromatin accessibility and looping, it is not clear what is meant by "shifts" or "increased/decreased" interactions because the correlations being described don't have an intrinsic increase/decrease direction (as might be assigned with a genotype). Would be better to describe the relationships in terms of positive or negative correlations.

**Reviewer 3**

**Were you able to assess all statistics in the manuscript, including the appropriateness of statistical tests used?**

Yes, and I have assessed the statistics in my report.

**Comments to author:**

Shi et al. have performed a very relevant work that will be of interest for the community and that also presents a consistent analysis framework that might be implemented in order diseases and cellular contexts. The conclusions derived from the study results are clear and properly supported by evidence and the manuscript is clear and well-written. Nevertheless, There are some points that need to be addressed before considering this manuscript for publication:

MAJOR POINTS

1) It should be clerly stated how many samples per cell type and sample type remained in the final analysis and how many of them were obtained from the same donor, as it is not clear neither in the manuscript text, nor in the tables or in the figures.

2) In the text it is stated "We identify 69,303, 72,884, 30,415 and 12,000 loops from merged maps from CD4+, CD8+, CD8+ synovial fluid and CD4+ synovial fluid T cells respectively". This QTL number differences should be justified, for example, what was the statistical power to detect QTLs in each layer?

3) Authors used the Pearson correlation coeficient to analyze the relationship between loops and gene expression, did they try different analyses based on Bayesian strategies, such as COLOC, to support colocalization between different types of QTLs?

4) It is reported that "about 20% of GWAS loci have one variant overlapping a CTCF peak", is this an expected proportion or are the GWAS loci enriched in CTCF peaks?

5) Several eQTLs are established based on the "Public eQTL" dataset, for example rs4409785 was associated with a reduction of expression of SESN3, why didn't the authors use the generated disease-associated eQTL dataset? Are the individuals included in the "Public eQTL" dataset healthy or affected?

6) Were any differences in interaction or compacteness between blood CD4+ and synovial CD4+ cells observed?

7) The generation of a queryable repository with a visualization interface would greatly improve the impact of the generated dataset in the scientific community.

MINOR POINTS

1) In the text it is stated "As expected, 75% of these loops overlap a CTCF peak in at least one anchor, with stronger loops more likely to overlap a CTCF peak, although only 42% overlap a CTCF peak at both anchors (Supplementary Figure 2A-C)." However, this percentages are not easy to infer from the figures. Authors should consider other representations, for example, density distribution or histograms.

2) Did the authors check the effects of the analysed variants in publicly accesible dataset in scarce CD4+ cell subtypes such as regulatory T cells?

3) What is the meaning of "differentially bound ATAC-seq peaks"? Differentially accesible chromatin regions?

4) Several statements such as "we could recover 38% of caQTL that directly overlap their feature, but caQTL could only recover 13% of significant allelic imbalance variants." are found in the text. Do the authors mean that the QTLs overlapped with other QTLs or other features?

5) Some FDR corrrected p-values reach FDR = 0. This is not correct and these p-values should be reported as FDR < 1/number of permutations.

6) T cells were separated based only on the presence of CD4 and CD8. However, several T cell subsets with very different characteristics are known. This reviewer is aware of the economic and logistic limitations of every study, but if the authors didn't consider subtype specific effects this should be discussed as a limitation of the study.

**Authors Response**

**Point-by-point responses to the reviewers’ comments:**

## Reviewer 1

This study investigates the interplay among gene expression, chromatin accessibility, and 3D genome organization in CD4+ and CD8+ primary T cells from patients with psoriatic arthritis (PsA) and healthy controls. The authors have generated a vast amount of data, including 108 Hi-C libraries with 49B read depths, to link genetic variants to the variability of gene expression, chromatin accessibility, and 3D genome organization. However, despite the valuable resources provided, the authors fail to provide a well-confirmed conclusion. As acknowledged by the authors themselves, the variability originating from sample heterogeneity, experimental variations, and analysis limitations outweighs the biological variability targeted in this study. Thus, the authors should reposition this valuable resource to reach more robust conclusions. Below are more specific comments:

1.            The figure resolution is too low to interpret the results. The authors should provide high-resolution images.

We have produced new high-resolution figures with larger, easier to read text.

2.            Detailed clinical information about the patients should be provided to enhance the understanding of the results. The clinical information for the samples collected in this study was not accessible from the link provided by the authors.

The metadata was located within the subfolder PsA_cleaned_analysis/metadata/. However, we understand that this is an important piece of information, so we have moved it to the root directory, as well as attaching a copy on the submission as a supplementary table (Supplementary table 1).

3.            The authors should confirm the expression levels of marker genes in both patients and controls for each cell type. Also, the authors should examine the variability of the maker gene expression for each cell type and investigate whether such variation indicates the presence of potential unknown confounding factors.

As mentioned in the methods, we have used CD8 and CD4 expression, accessibility and looping to control for potential sample mislabelling. We have added these plots to the supplementary figures. In terms of confounding factors, we agree that, as indicated by another reviewer, it is likely that different individuals would have different cell subtype composition, as our isolation method does not target a specific subpopulation of CD8+ T cells or CD4+ T cells. Most of the analysis however are done using matched samples, so that the cell type composition between the three modalities should be identical.

4.            The authors should provide results on the reproducibility of the findings for selected samples. It is challenging to determine whether the heterogeneity issue in the multi-omics results reflects sampling heterogeneity, patient heterogeneity, or individual heterogeneity. At least the authors should collect samples twice from the same patients and determine the degree of reproducibility.

Unfortunately, due to the study design it was not possible to collect samples from the same patient multiple times. We have however generated a few replicate libraries for a subset of the samples. We have calculated Euclidean distance between samples, by first gathering the counts for peaks, loops strength and gene expression, running a PCA and then calculating the distance on the top 10 components, and we can show that Hi-C and RNA-seq libraries generated from the same sample have a smaller distance than different libraries (supplementary figure 2). For ATAC-seq unfortunately we only had 2 samples with replicate libraries, however their quality was particularly low. In that case we have also compared the distances between samples of the same cell type and all others, and the distances within the same cell type show smaller distances (supplementary figure 2).

5.            The authors essentially stratified the analysis in terms of active vs. non-active disease and active disease vs. controls. However, given the identified limited number of differentially expressed genes, the authors should clarify why such a comparison is biologically meaningful.

**Although we had limited statistical power to detect significant differences, disease activity is an important score in Rheumatology clinics. It is used routinely to monitor patients, assess treatment response and inform disease management decisions. Therefore, we attempted this analysis with the aim to identify biomarkers that may have potential clinical significance.**

6.            To demonstrate their results clearly, the authors need to perform clustering analysis of gene expression data and identify subpopulations based on molecular phenotypes (i.e., gene expression). Subsequently, for each group, the authors need to integrate chromatin accessibility and Hi-C results to explain the variation in gene expression patterns within each group. Without such clear stratified analyses for each omics result, it is hard to justify the rationale for the integration of multi-omics results.

**We have generated clusters on gene expression data (Supplementary figure 7). We conducted differential expression analysis to identify genes and pathways driving differences between clusters. To integrate chromatin accessibility, we selected clusters of interest where differences between them were driven by immune related pathways and conducted a differential accessibility analysis (clusters 1 vs 2 and 1 vs 3 for CD4+ T-cells, and clusters 1 vs 2 for CD8+ T-cells). We show that there are distinct chromatin accessibility patterns between clusters that correlate with gene expression clusters (supplementary figure 9).**

7.            The authors may have reached overly conclusive statements due to limited data analysis. For example, the authors concluded that 'the way genetic variants affect these interactions is not primarily driven by alteration of the CTCF binding.' However, without CTCF ChIP-seq data for each individual, it is difficult to determine whether alteration of CTCF binding is a factor driving 3D genome variability.

**We agree with the reviewer and have now softened this statement “the way genetic variants affect these interactions may not always be driven by alteration of the CTCF binding”.**

8.            The authors suggest several GWAS loci and how the variants potentially involve pathogenic gene regulation in the context of chromatin accessibility and chromatin loops, but without minimum experimental validation using genome editing, it is challenging to confirm such conclusions. The authors should present at least one experimentally validated example linking GWAS variants to molecular phenotypes.

**We have performed experimental validation using CRISPR/Cas9 genome editing for the *ACOXL/BCL2L11* locus. We show that, after stimulation, primary CD4+ T-cells lacking the enhancer containing the disease associated SNP rs13401811 show decreased expression of *BCL2L11* compared to wild type T-cells. We have added this experiment to the manuscript (see results, methods and supplementary figures 12 and 16).**

## Reviewer 2

The authors of this paper have generated a nice dataset of Hi-C, ATAC-seq and RNA-seq from primary CD4+ and CD8+ T cells isolated from peripheral blood and synovial fluid of psoriatic arthritis patients and normal individuals. The Hi-C data in particular are a unique resource, that is far larger than prior Hi-C studies in primary cells. The dataset is a major strength of the paper. The Hi-C data allows the authors to detect the effects of gene expression and chromatin QTLs are chromatin architecture at a high resolution. There are some good examples of this shown in the paper, such as at the IKZF3/GSDMB/ORMDL3 locus. There are several weaknesses in the paper. A few more QC steps should be taken. The QTL analyses have a several issues, including possibly incorrectly applied thresholds for statistical significance and multiple testing correction. These issues are described in detail below.

Major Comments:

1) One RNA-seq sample is likely to be mislabeled as CD4 patient that is actually CD8, based on UMAP shown in Fig 1B.  Suggest checking expression of CD4/CD8 specific marker genes and reassigning sample label if necessary.

**As also shown in the response to the first question by Reviewer 1, we have used CD8 and CD4 expression, accessibility and looping to control for potential sample mislabelling. The sample pointed out here was indeed mislabelled and has been now reassigned correctly.**

2) Related to the above point, I suggest checking RNA-seq/Hi-C/ATAC-seq samples are concordant with genotyping from SNPChip. In most large studies like this one, there are a few sample mixups where the genotypes don't match the assays. These can be resolved by looking at pairwise correlations between assay genotypes. One tool to do this is NGSCheckMate.

**We agree with the reviewer, and we had already performed the suggested analysis using the QTLtools v1.3.1 mbv function in the original version of the manuscript.**

**Related to this and the previous point, and also a comment by Reviewer 1, we have now modified the following paragraph in the first section of the methods “To confirm that there was no sample mislabelling the concordance of the genotype with the bam file was checked using the QTLtools v1.3.1 mbv function (60). To identify mismatches between CD4+ T cells and CD8+ T cells we used the loop strength of the loops surrounding the CD4 and CD8A genes for the Hi-C data (Supplementary figure 13A-B), the gene expression of the CD4 and CD8A genes for the RNA-seq data (Supplementary figure 13C-D), the chromatin accessibility of the promoters of CD4 and CD8A for ATAC-seq (Supplementary figure 13E-F )., and the loop strength of the loops surrounding the CD4 gene for the Hi-C data. Sample mismatches were then corrected in the metadata files.”**

3) Additional QC of the ATAC-seq data should be performed. ATAC-seq quality can vary dramatically, so it is important to calculate the fraction of reads in peaks (FRiP) and TSS enrichment score for each sample. Samples that do not meet QC thresholds should likely be removed from the study. ENCODE has some guidelines on this ([https://urldefense.com/v3/__https://www.encodeproject.org/atac-seq/__;!!PDiH4ENfjr2_Jw!GHmYa-x5dBRlkZLjvisQkvSvOM6J1S-EOz69mL1CgfltkckB8v73yM9x248p5i14uXyx4-EyHKdA4ealq4drvlRoZg4$](https://urldefense.com/v3/__https:/www.encodeproject.org/atac-seq/__;!!PDiH4ENfjr2_Jw!GHmYa-x5dBRlkZLjvisQkvSvOM6J1S-EOz69mL1CgfltkckB8v73yM9x248p5i14uXyx4-EyHKdA4ealq4drvlRoZg4$)[encodeproject[.]org]) although their minimum thresholds may be too strict for primary cells, which generally give lower quality data than cell lines.

**We had performed the QC suggested for the ATAC-seq data but did not include it in the original version of the manuscript. We thank the reviewer for pointing out this omission and have now added Fraction of reads in peaks (FRiP) and Fraction of reads in TSS (FRiTSS) for all ATAC-seq samples to the hosted directory and in the revised manuscript (Supplementary figure 14).**

4) Table 2 describes total number of insulation score QTLs (insQTL), loop strength QTLs (loopQTL), gene expression QTLs (eQTLs) and chromatin accessibility QTLs (caQTLs). It's not clear what the sample sizes are, and I would suggest adding this to the table. I would guess that the sample sizes are all 74 (n=55 PsA patients + n=19 healthy controls) but it's not obvious that this is the case or if the numbers are consistent across assays/cell types.

**Sample sizes were added to table 2.**

5) The numbers of reported significant QTLs seems too high given sample sizes. The number of eGenes seems on the high side (see Fig 2A in the GTEx paper for comparison [https://urldefense.com/v3/__https://pubmed.ncbi.nlm.nih.gov/32913098/__;!!PDiH4ENfjr2_Jw!GHmYa-x5dBRlkZLjvisQkvSvOM6J1S-EOz69mL1CgfltkckB8v73yM9x248p5i14uXyx4-EyHKdA4ealq4dr3z2ukuo$](https://urldefense.com/v3/__https:/pubmed.ncbi.nlm.nih.gov/32913098/__;!!PDiH4ENfjr2_Jw!GHmYa-x5dBRlkZLjvisQkvSvOM6J1S-EOz69mL1CgfltkckB8v73yM9x248p5i14uXyx4-EyHKdA4ealq4dr3z2ukuo$)[pubmed[.]ncbi[.]nlm[.]nih[.]gov]). Based on my experience with ATAC-seq datasets of size n=100-150, the number of caQTLs identified seems much too high. I would expect the number of caQTLs identified to be about 10,000 or lower with this dataset (~5% of total peaks), rather than the reported 19-26k.

6) Because of the point above, and the methods section, I am concerned that an inappropriate p-value threshold has been used to determine significance.  The methods say: "To calculate corrected p-values, we performed a permutation pass with 1000 permutations. However, corrected p-values are only calculated on the top hit. We find that for all our modalities an equivalent uncorrected pvalue to a corrected p-value of 0.1 is approximately 1x10-4. For the calculation of the overlaps between the modalities, we decided to use this latter p-value as the top hit would rarely overlap between the different modalities."

Based on the above description, there appears to be some confusion about the nature of the p-values returned from QTLTools and how they should be used.  In QTL analysis, there is a multiple testing problem for each gene (or peak or loop) considered because many SNPs are tested for association with each gene (or peak). Because the tested SNPs are not independent (due to LD) a standard FDR correction may not be appropriate.  A per-locus p-value correction is typically done by performing permutations that maintain the correlations between nearby SNPs (LD). Adjusted p-values are then computed from permutations using the most significant p-value from each permutation, as described in the FastQTL paper. The adjusted p-value should be interpreted as a p-value for the gene (i.e. encompassing all of the SNPs that are tested). The statement that "the corrected p-values are only calculated on the top hit" is not totally correct—even though the top hit is used to estimate the corrected p-value in the permutations, the permutation p-value should not be viewed as assigned to a particular SNP--rather it is for the entire locus.  In other words, a low corrected p-value means that there is at least one significant association between genotypes of the SNPs at the locus and the expression of the gene. You can use the adjusted p-value to identify significant eGenes, and then use the most significant SNP as the lead SNP. (side note: if you want to identify more than one association per gene, then a conditional analysis needs to be performed (QTLTools has the ability to do this)).

The authors use a nominal p-value cutoff of 1e-4, which is claimed to be roughly equivalent to an adjusted p-value of 0.1 (it's also not appropriate to use an adjusted p-value of 0.1). First, I doubt that this approximation will hold for all loci, because the relationship between nominal p-values and the adjusted p-value depends on the number of SNPs tested at the locus and on LD. Second, while the adjusted p-value accounts for the number of SNPs tested for a given gene (or peak or loop) I don't think it accounts for the total number of genes tested (or peaks or loops). This becomes a larger problem when more elements are tested, such as in the case of peaks and loops.

I suggest that the authors should take the adjusted p-values output by QTLTools (the ones computed using the Beta distribution), and then compute an FDR across all tested genes (or peaks or loops) using the adjusted p-values to determine which genes have significant eQTLs. The same approach should be applied to the other QTL types. I also recommend that the authors make qqplots of -log10 observed vs. expected p-values so that the calibration of the p-values can be examined.

**We thank the reviewer for the thorough discussion presented in comments 5 and 6. We have now improved the QTL analysis as suggested by adding the additional FDR correction step, and updated results in the corresponding sections. In addition, QQ plots are now presented (Supplementary figure 15).**

7) The low concordance of overlaps of loopQTLs between CD4 and CD8 T cells is concerning and suggests that potentially most of the identified loopQTLs are false positives. This may relate to the use of inappropriate p-value thresholds discussed above rather than sensitivity as hypothesized by the authors.  It would be good to see how overlap changes as a function of significance.

**After performing the suggested modifications, the new concordances have been updated. As predicted by the reviewer, results have improved; in particular, the number of significant loopQTLs has reduced significantly.**

8) The overlaps analysis for eQTLs and caQTLs doesn't seem correct. The methods state "For overlaps between eQTLs and caQTLs, we matched the ATAC-seq peaks that overlapped the TSS sites for the genes in the eQTL dataset." Why should only peaks overlapping the TSS be used? Shouldn't it be possible to consider distal caQTLs for overlap with distal eQTLs?

**We focused this analysis on ATAC-seq peaks that overlapped the TSS for the eQTL genes to ensure that we are considering regulatory elements that are likely to regulate the expression of the gene in question. We felt like by looking at other ATAC-seq peaks representing distal enhancers, we cannot be confident that these are regulating that specific gene.**

9) The allelic imbalance analysis for both chromatin accessibility and chromatin loops uses a binomial test. The binomial test for allelic imbalance is known to give a lot of false positives due to overdispersion in genomic count data, and for this reason a beta-binomial test is generally used instead. Also, a two-sided test should be used, not a one-sided test.

**Results have been updated using the beta binomial test as implemented in apeglm, see updated methods. We have updated results using S-values. Please note that as a result some SNPs are not significant anymore and have removed mentions of these in the updated version of the manuscript.**

10) The analysis of the overlap between variants and CTCF binding sites is difficult to interpret without some information about the null expectation. For example, for the statement "15% of loops with allelic imbalance have one variant located within a CTCF peak", is this an enrichment over what is expected by chance?

**This section has now been updated with new numbers after revising the QTL analysis as suggested by the reviewer. In addition, focusing on disease risk loci (see also Reviewer 3’s point number 4), we have tested formally whether GWAS variants are enriched in CTCF peaks using fGWAS, for several autoimmune diseases where T cells play an important role and for which summary statistics were available (PsA, RA, JIA). This analysis indicated no significant enrichment.**

Minor Comments:

11) I strongly suggest putting more information about the number of samples, type of samples and cell types in the abstract of the paper. The dataset is a really important aspect of the paper, but currently it is not described until the results section.

**We have now added this important information in the abstract**.

12) "with the synovial fluid samples forming a separate cluster from the peripheral blood samples" Suggest mentioning that T cells were obtained from peripheral blood and synovial fluid prior to this sentence.

**The sentence has been modified as suggested.**

13) P6, L33: following reference to "Supplementary Figure 5" there is an error message in the PDF:: "Error! Reference source not found"

**This typo has been corrected.**

14) P6, L40-42: Differences between CD8+ blood / synovial are described but what about CD4+ cells? They seem left out here.

**Unfortunately, we only managed to obtain one CD4+ T-cell sample from synovial fluid and therefore it was not possible to analyse differences in blood vs synovial fluid for this cell type.**

15) Fig 1F: shows correlation between gene expression and insulation score. Legend states that each dot is a gene, but then there are a fairly small number of genes included in this plot. The legend should state which subset genes are used here, since doesn't appear to be all genes.

**Only genes that had some differential expression between CD4 and CD8 were tested. Figure legend has been updated accordingly.**

16) P7: Throughout the paragraph on correlations between chromatin accessibility and looping, it is not clear what is meant by "shifts" or "increased/decreased" interactions because the correlations being described don't have an intrinsic increase/decrease direction (as might be assigned with a genotype). Would be better to describe the relationships in terms of positive or negative correlations.

**We have now used the wording suggested by the reviewer in this section.**

## Reviewer 3

Shi et al. have performed a very relevant work that will be of interest for the community and that also presents a consistent analysis framework that might be implemented in order diseases and cellular contexts. The conclusions derived from the study results are clear and properly supported by evidence and the manuscript is clear and well-written. Nevertheless, there are some points that need to be addressed before considering this manuscript for publication:

MAJOR POINTS

1) It should be clearly stated how many samples per cell type and sample type remained in the final analysis and how many of them were obtained from the same donor, as it is not clear neither in the manuscript text, nor in the tables or in the figures.

**This point was also raised by reviewer 1 in their first comment. We have added a supplementary table (supplementary table 1) with detailed donor information and have added sample sizes to Table 2.**

2) In the text it is stated "We identify 69,303, 72,884, 30,415 and 12,000 loops from merged maps from CD4+, CD8+, CD8+ synovial fluid and CD4+ synovial fluid T cells respectively". This QTL number differences should be justified, for example, what was the statistical power to detect QTLs in each layer?

**We would like to point out that this does not refer to QTLs, but to the number of significant loops identified for each cell type. The loop calling method utilised is sensitive to the sequencing depth; since some cell types had more samples (for example, we had limited numbers of T cells isolated from synovial fluid compared to T cells from blood), total depth was higher and therefore more significant loops were identified.**

3) Authors used the Pearson correlation coefficient to analyse the relationship between loops and gene expression, did they try different analyses based on Bayesian strategies, such as COLOC, to support colocalization between different types of QTLs?

**Unfortunately, we believe sample size was not sufficient for that the type of analysis suggested.**

4) It is reported that "about 20% of GWAS loci have one variant overlapping a CTCF peak", is this an expected proportion or are the GWAS loci enriched in CTCF peaks?

**We tested for enrichment of GWAS variants in CTCF peaks using fGWAS, for a number of autoimmune diseases where T cells play an important role and for which summary statistics were available (PsA, RA, JIA). This analysis indicated no significant enrichment. We have presented this new analysis in the results and Supplementary table 3.**

5) Several eQTLs are established based on the "Public eQTL" dataset, for example rs4409785 was associated with a reduction of expression of SESN3, why didn't the authors use the generated disease-associated eQTL dataset? Are the individuals included in the "Public eQTL" dataset healthy or affected?

**The public dataset used was from the eQTLgen repository, which is the largest blood derived eQTL meta-analysis available, including 31,684 individuals. It includes predominantly data from healthy individuals, but it also contains a limited number of disease cohorts such as heart disease. Our eQTL dataset was significantly smaller than eQTLgen, and therefore had less power to detect significant eQTLs. In particular, rs4409785 was not significantly correlated with the expression of *SESN3* in our cohort.**

6) Were any differences in interaction or compacteness between blood CD4+ and synovial CD4+ cells observed?

**As stated in response to Reviewer 2’s question number 14, unfortunately, we only had access to one CD4+ T-cell sample from synovial fluid and therefore it was not possible to analyse differences in blood vs synovial fluid for this cell type.**

7) The generation of a queryable repository with a visualization interface would greatly improve the impact of the generated dataset in the scientific community.

**We have made available a script that allows the community to explore all the results, which includes an updated guide to help users. It is available at https://colab.research.google.com/github/ChenfuShi/PsA_cleaned_analysis/blob/main/integration_analysis/Everything_printer_public.ipynb**

MINOR POINTS

1) In the text it is stated "As expected, 75% of these loops overlap a CTCF peak in at least one anchor, with stronger loops more likely to overlap a CTCF peak, although only 42% overlap a CTCF peak at both anchors (Supplementary Figure 2A-C)." However, this percentages are not easy to infer from the figures. Authors should consider other representations, for example, density distribution or histograms.

**Please note that the figure is not trying to show those percentages but rather that loops that overlap CTCF have stronger local enrichment. We have moved the mention to the figure to clarify this.**

2) Did the authors check the effects of the analysed variants in publicly accesible dataset in scarce CD4+ cell subtypes such as regulatory T cells?

**eQTL data for CD4+ T cells is available as part of the DICE database** <https://dice-database.org/>**. However, the sample size of this dataset is modest compared to eQTLgen, and therefore, we did not identify any novel correlations that had not already been found in eQTLgen.**

3) What is the meaning of "differentially bound ATAC-seq peaks"? Differentially accesible chromatin regions?

**We have replaced “differentially bound” with “differentially accessible”.**

4) Several statements such as "we could recover 38% of caQTL that directly overlap their feature, but caQTL could only recover 13% of significant allelic imbalance variants." are found in the text. Do the authors mean that the QTLs overlapped with other QTLs or other features?

**Please note that, in response to Reviewer 2’s comments, we have substantially updated the QTL analysis since we acknowledge lack of FDR correction. As a result, these statements have been updated.**

5) Some FDR corrrected p-values reach FDR = 0. This is not correct and these p-values should be reported as FDR < 1/number of permutations.

**We have corrected this to refer to the minimum positive nonzero value in python which is 1e-308.**

6) T cells were separated based only on the presence of CD4 and CD8. However, several T cell subsets with very different characteristics are known. This reviewer is aware of the economic and logistic limitations of every study, but if the authors didn't consider subtype specific effects this should be discussed as a limitation of the study.

**We agree with the reviewer and have added a comment on this limitation in the discussion.**

**Second round of review**

**Reviewer 1**

The authors have made substantial progress in addressing my previous comments, but there are areas where further revisions would be beneficial. There are still some issues with labeling, such as the y-axis in panels S13C and S13D, which seems to represent gene expression. Additionally, the manuscript would benefit from a more comprehensive revision to enhance clarity. Several figures also deviate from standard guidelines, which impacts the interpretability of the results. It would be helpful if the response letter included specific details on the changes made and any new analyses conducted in the revised manuscript. Below you can find more specific explanations for my previous comments.


1. Regarding Previous Comment 3: If the authors cannot rule out that variations in marker gene expression may be due to individual-specific heterogeneous cellular composition, how can they be confident that genetic variations are responsible for variability in gene expression, chromatin accessibility, or chromatin loops, rather than simple variations in cellular heterogeneity?

2. On Reproducibility: The authors have reported Euclidean distances, suggesting that the distance between replicates is relatively shorter compared to the distance between individuals. However, to substantiate reproducibility claims, the authors should present direct hierarchical clustering results based on Hi-C and RNA-seq data and test whether replicates are clustered together. Relying solely on Euclidean distance distributions is insufficient evidence for reproducibility.

3. Stratified analyses: The clustering analysis based on gene expression (Fig. S7) does not show a clear correlation with disease status. This suggests that the samples may not be appropriately categorized by disease status or that the samples are too heterogeneous to distinguish between healthy and diseased states. Furthermore, the presentation of differential accessible peaks as a heatmap (Fig. S9) involves defining differential peaks between groups and then displaying them in a heatmap, which may constitute circular logic. To establish that differentially accessible peaks regulate cluster-specific differential gene expression, the authors should independently identify differential peaks and genes and test whether the cluster-specific peaks and genes are linked through chromatin contacts.

4. Experimental Validation of GWAS Loci: While the effort to validate GWAS loci using genome editing is appreciated, the revised manuscript predominantly discusses well-known GWAS-SNP target genes. To enhance the novelty of the study, it would be valuable to perform similar experiments targeting novel relationships leveraged by the large-scale dataset, which is emphasized in the abstract. Simply confirming known relationships does not sufficiently demonstrate the novelty of the findings. Additionally, the inclusion of nearby genes as controls is required.

**Reviewer 2**

The authors have done a great job at addressing the comments from the previous review. The QTL and allele specific analysis in particular are greatly improved. I have a couple of minor suggestions:

1) Abbreviations (e.g. GWAS, RA, PsA) should be defined on first usage in the abstract
2) I suggest removing the following sentences that were added to the revised manuscript. I feel they have the effect of underselling the findings and I am not sure they add anything useful. I am not totally sure what is meant by the limited sensitivity. I agree these is probably limited power to detect loop QTLs, but a large number of chromatin accessibility QTLs are detected (in line with prior caQTL studies):
“Additionally, these results indicate that, whilst our study is the largest of its kind for chromatin conformation and is among the largest for chromatin accessibility QTLs, the sensitivity remains significantly limited. Additional information can be found in the methods.”

**Reviewer 3**

I consider that the authors have greatly improved the QTL analysis and that they addressed properly my concerns.

**Authors Response**

**Point-by-point responses to the reviewers’ comments:**

**Reviewer #1**

2. On Reproducibility: The authors have reported Euclidean distances, suggesting that the distance between replicates is relatively shorter compared to the distance between individuals. However, to substantiate reproducibility claims, the authors should present direct hierarchical clustering results based on Hi-C and RNA-seq data and test whether replicates are clustered together. Relying solely on Euclidean distance distributions is insufficient evidence for reproducibility.
*We would like to clarify that, due to the study design, it was not possible to collect samples from the same patient multiple times, which means we did not have true replicates. Therefore, our capacity to test reproducibility formally is limited, unfortunately.
For a very small subset of samples, sequencing was repeated due to substandard quality of the original data. For our limited Hi-C replicates, direct hierarchical clustering of the previously reported Euclidean distances was performed using Ward’s linkage method (as implemented in scipy.cluster.hierarchy), and the resulting dendrogram demonstrates that replicate Hi-C libraries cluster closely, confirming the sensitivity of our approach in distinguishing between individual samples (Supplementary figure 2B).
After revisiting QC metrics, we have decided to exclude the RNA-seq replicate libraries from this analysis, due to poor quality of the original libraries.*


3. Stratified analyses: The clustering analysis based on gene expression (Fig. S7) does not show a clear correlation with disease status. This suggests that the samples may not be appropriately categorized by disease status or that the samples are too heterogeneous to distinguish between healthy and diseased states. Furthermore, the presentation of differential accessible peaks as a heatmap (Fig. S9) involves defining differential peaks between groups and then displaying them in a heatmap, which may constitute circular logic. To establish that differentially accessible peaks regulate cluster-specific differential gene expression, the authors should independently identify differential peaks and genes and test whether the cluster-specific peaks and genes are linked through chromatin contacts.
*As suggested by the reviewer, we have independently identified differentially expressed genes and differentially accessible peaks across the clusters presented in Supplementary figure 9, which were defined by gene expression, and checked whether they are preferentially linked through chromatin looping. Cluster specific chromatin loops were generated using Mustache. Within each cluster, we found that the number of significant interactions between genes and differentially accessible peaks was significantly higher for differentially expressed genes compared to non-differentially expressed genes (Supplementary figure 10). We performed this analysis for clusters across both cell types (CD4 and CD8 T-cells). For CD4+ T-cells, there were three clusters but one of the clusters’ sample size (cluster 2) was too small to perform this analysis (n=2).*


**Reviewer #2:**

1) Abbreviations (e.g. GWAS, RA, PsA) should be defined on first usage in the abstract
*We thank the reviewer for spotting these mistakes; abbreviations have now been defined on first usage in the abstract.*

2) I suggest removing the following sentences that were added to the revised manuscript. I feel they have the effect of underselling the findings and I am not sure they add anything useful. I am not totally sure what is meant by the limited sensitivity. I agree these is probably limited power to detect loop QTLs, but a large number of chromatin accessibility QTLs are detected (in line with prior caQTL studies):
“Additionally, these results indicate that, whilst our study is the largest of its kind for chromatin conformation and is among the largest for chromatin accessibility QTLs, the sensitivity remains significantly limited. Additional information can be found in the methods.”
*We agree with the reviewer and have now removed the above-mentioned sentences.*
